# KDM5B cooperates with CRL4B complex to promote the tumorigenesis of ER+ breast cancer via regulating cholesterol metabolism

**DOI:** 10.1038/s41419-026-08438-1

**Published:** 2026-02-07

**Authors:** Yunkai Yang, Tianyang Gao, Baowen Yuan, Xinhui Hao, Miaomiao Huo, Ting Hu, Tianyu Ma, Min Zhang, Die Zhang, Xu Teng, Hefen Yu, Wei Huang, Jingyao Zhang, Yan Wang

**Affiliations:** 1 State Key Laboratory of Molecular Oncology, National Cancer Center/National Clinical Research Center for Cancer/Cancer Hospital, Chinese Academy of Medical Sciences and Peking Union Medical College, Beijing, China; 2 Department of Biochemistry and Molecular Biology, School of Basic Medical Sciences, Capital Medical University, Beijing, China; 3 Institute of Cancer Research, Henan Academy of Innovations in Medical Sciences, Zhengzhou, Henan China

**Keywords:** Breast cancer, Cancer stem cells

## Abstract

Estrogen receptor-positive (ER+) breast cancer is the predominant subtype of breast cancer, and its development is closely linked to metabolic reprogramming, including alterations in cholesterol metabolism. Therefore, this study aimed to investigate the functional interplay between lysine demethylase 5B (KDM5B) and the Cullin-RING ligase 4B (CRL4B) complex in modulating cholesterol metabolism to promote ER+ breast cancer progression. Immunohistochemical assays and bioinformatic analysis of various cancer databases were performed to examine KDM5B expression levels in breast cancer. Additionally, KDM5B overexpression and knockdown were performed to investigate the role of KDM5B in breast cancer cell proliferation and progression. Notably, we identified physical and functional interactions between KDM5B and the CRL4B subunits, CUL4B and DDB1. Mechanistically, KDM5B recruits CRL4B to the promoters of INSIG1 and INSIG2, which are key regulators of cholesterol biosynthesis and uptake, and suppresses their expression by upregulating H2AK119ub1 and downregulating H3K4me3 histone marks, thereby promoting the proliferation, migration, and invasion of tumor cells. Functional assays revealed that disruption of the KDM5B–CRL4B axis impairs cholesterol homeostasis and inhibits tumor growth. KDM5B upregulation was significantly negatively correlated with the survival rates in various cancer types, including thyroid, lung, esophageal and colorectal cancers. Overall, these findings establish a novel regulatory axis in cholesterol metabolism, uncover potential therapeutic vulnerabilities in ER+ breast cancer, and suggest that targeting the KDM5B could provide a strategy to curb tumor progression.

## Introduction

KDM5B, a member of the JmjC domain-containing histone demethylases, is a critical epigenetic regulator that specifically erases di- and tri-methyl groups from H3K4me2/3 [1–3]. This activity represses gene transcription and is vital for controlling cellular differentiation [4, 5], proliferation [6], and stress responses [7, 8]. Dysregulation of KDM5B has been implicated in various cancers, where it often functions as an oncogene by altering chromatin states and transcriptional networks critical for tumor development [9–11]. KDM5B is frequently overexpressed in breast cancer, particularly in ER+ subtypes [12]. Moreover, KDM5B overexpression correlates with poor prognosis [13], tumor progression [12, 13], and therapy resistance [14–16]. However, that KDM5B may activate transcription has also been reported in other studies [17, 18]. Given these inconclusive results, the mechanistic role of KDM5B in cancer requires further investigation.

CUL4B, DDB1, and ROC1 are core components of the CRL4B complex, an E3 ubiquitin ligase that regulates various cellular processes by targeting specific substrates for ubiquitination and subsequent proteasomal degradation. For example, the CRL4B complex suppresses Th1 and Th2 cell differentiation through histone H2AK119 mono-ubiquitination [19]. CRL4B physically associates with the SIN3A-HDAC complex to drive cell cycle progression [20]. In cancer, CUL4B and DDB1 work synergistically within the CRL4B complex to interact with SIRT1 or polycomb repressive complex 2 (PRC2) to promote tumorigenesis by repressing the expression of tumor suppressor genes [21, 22]. Importantly, dysregulation of CUL4B and DDB1 leads to aberrant protein turnover, including tumorigenic phenotypes such as uncontrolled proliferation, disrupted circadian rhythms, enhanced DNA damage tolerance, and metabolic adaptation [22–24]. Although KDM5B and CRL4B have been independently linked to cancer progression, their potential functional cooperation remains unclear.

Cholesterol plays a vital role in cellular processes such as maintaining membrane integrity, steroid hormone synthesis, and intracellular signaling. Dysregulated cholesterol metabolism has emerged as a hallmark of cancer, contributing to tumorigenesis and cancer progression [25]. Cholesterol homeostasis is frequently altered in cancer cells, with increased cholesterol biosynthesis and uptake, and reduced efflux, facilitating rapid cell proliferation and survival under stress conditions [26, 27]. Cholesterol metabolism intersects with the metabolic reprogramming of cancer by providing precursors for steroid hormones and bile acids, which are critical in hormone-driven cancers such as breast and prostate cancer [26]. In ER+ breast cancer, cholesterol-derived metabolites such as 27-hydroxycholesterol act as selective estrogen receptor modulators (SERMs), promoting tumor growth [28, 29]. Moreover, excess cholesterol supports oxidative stress resistance and energy production via mitochondrial adaptation, further enhancing the survival of tumor cells [30]. Emerging evidence suggests that cholesterol metabolism is tightly regulated at both the transcriptional and epigenetic levels [31]. However, regulatory networks that integrate epigenetic modifiers and metabolic pathways are poorly characterized.

In this study, we systematically investigated the pathophysiological roles and molecular mechanisms of KDM5B in the development and progression of ER+ breast cancer. Specifically, we explored the interplay between KDM5B and the CRL4B complex in regulating cholesterol metabolism and their cooperative contribution to the promotion of ER+ breast cancer proliferation, invasion, and stemness.

## Results

### KDM5B acts as an oncogene in breast cancer, especially in the ER+ subtype

To examine the role of KDM5B in breast cancer, we compared KDM5B expression levels in breast cancer and normal tissues using TCGA and several GEO datasets. KDM5B expression was significantly higher in breast cancer tissues (18 paired breast cancer specimens and adjacent normal tissues) than in normal tissues (Fig. S1A, B). Similarly, KDM5B protein expression was consistently higher in breast cancer tissues than in normal tissue (Fig. S1C). Notably, KDM5B expression was positively correlated with breast cancer stages (Fig. S1D) and negatively correlated with the survival probability of patients with breast cancer (Fig. S1E, F). High KDM5B expression was an independent predictor of low survival rates in patients with breast cancer undergoing chemotherapy or endocrine therapy (Fig. S1G).

**Fig. 1:**
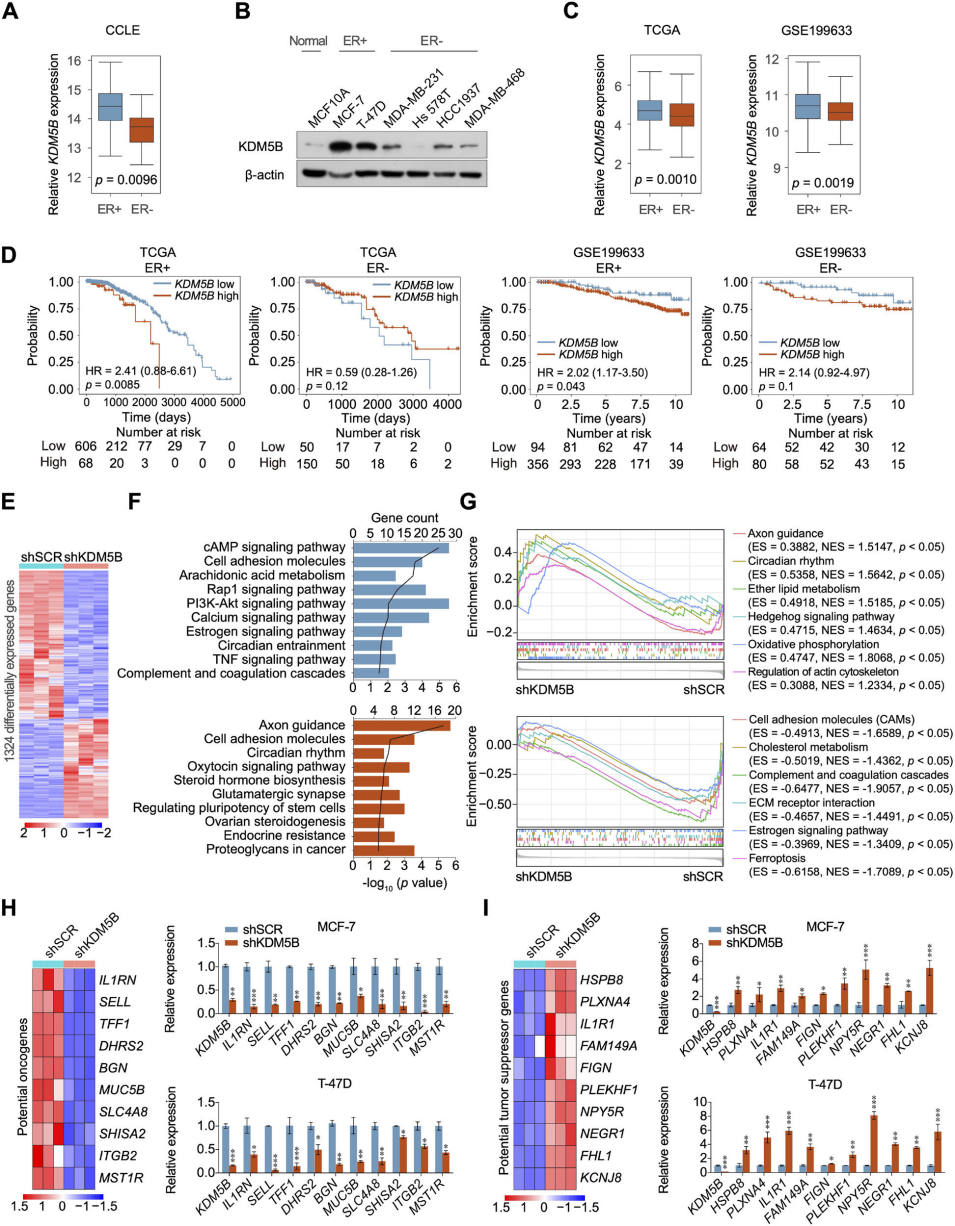
KDM5B was upregulated and predicted poor prognosis in ER+ breast cancer cells. **A** The KDM5B levels were compared between ER+ and ER- breast cancer cell lines using CCLE dataset. **B** The protein levels of KDM5B in breast cancer cells were detected by western blot. β-actin was served as a loading control. **C**, **D** The correlation between KDM5B expression and patient prognosis was analyzed using TCGA and GSE199633 datasets in ER+ and ER- breast cancer. **E** Heatmap depicting the expression profile changes in shSCR and shKDM5B MCF-7 cells. **F** KEGG analysis of DEGs in shCR and shKDM5B MCF-7 cells. Data were analyzed using DAVID (https://david.ncifcrf.gov/). **G** GSEA analysis of DEGs in shCR and shKDM5B MCF-7 cells. GSEA analysis was performed using Sangerbox tools (http://vip.sangerbox.com/). **H**, **I** RT-qPCR was performed to validate the RNA-seq results. The expression of several potential oncogenes and TSGs was detected in shSCR and shKDM5B ER+ breast cancer cells. ACTB was used as an internal control. Data were shown as mean ± SD. The data were analyzed by two-tailed unpaired *t*-test. *p < 0.05, **p < 0.01, ***p < 0.001.

KDM5B is a luminal lineage-driving oncogene in breast cancer [13]. Expectedly, KDM5B expression was higher in ER+ than in ER- breast cancer cell lines (Fig. 1A). Similarly, western blotting revealed that KDM5B protein levels were higher in ER+ than in ER- breast cancer cells (Fig. 1B). Analyses of the TCGA and GEO datasets demonstrated that ER+ breast cancer exhibited higher expression of KDM5B (Fig. 1C). Moreover, KDM5B expression was significantly correlated with the survival rate of patients with ER+ breast cancer (Fig. 1D). According to these results, we performed RNA-sequencing analysis in MCF-7 breast cancer cells acquiring KDM5B knockdown (Fig. 1E). The differentially expressed genes (DEGs) were enriched in multiple signaling pathways, including PI3K-AKT signaling, circadian rhythm, steroid hormone biosynthesis and pluripotency of stem cells (Fig. 1F). Additionally, GSEA revealed that KDM5B is involved in circadian rhythm, estrogen, ferroptosis and cholesterol metabolism signaling pathways (Fig. 1G). To validate the RNA-sequencing results, we selected several DEGs, including upregulated and downregulated genes, and measured their mRNA levels in ER+ breast cancer cells following KDM5B knockdown (Fig. 1H, I). The expression of these selected genes was also confirmed using the TCGA expression profile, which demonstrated that these genes might serve as potential oncogenes or tumor suppressors (Fig. S2A, B). Overall, these results demonstrate that KDM5B is an oncogene in breast cancer, specifically in ER+ breast cancer.

### KDM5B promotes the proliferation, invasion, and stemness of ER+ breast cancer cells

**Fig. 2:**
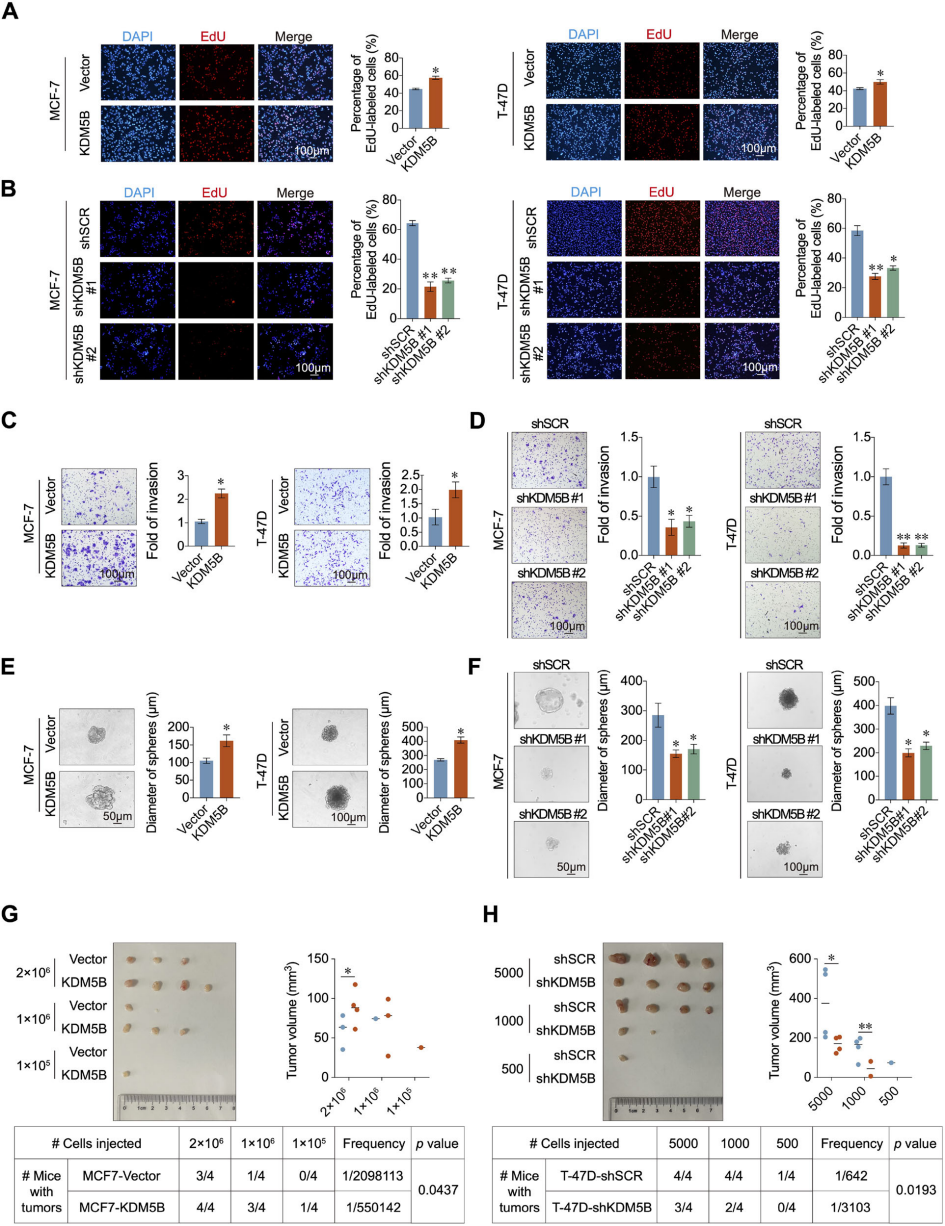
KDM5B promoted the proliferation, invasion and stemness of ER+ breast cancer cells. **A**, **B** EdU asays were performed to evaluate the growth ability of MCF-7 and T-47D cells acquiring KDM5B knockdown or overexpression. **C**, **D** Transwell asays were performed to evaluate the invasive ability of MCF-7 and T-47D cells acquiring KDM5B knockdown or overexpression. **E**, **F** The sphere formation assays were used to determine the influence of KDM5B on stemness in MCF-7 and T-47D cells with KDM5B-knockdown or -overexpression. **G** The tumor formation ability using limiting dilution assay was performed in Vecter or KDM5B-infected MCF-7 cells. The tumor volume was calculated, and the frequency of tumor formation was determined by ELDA. **H** The tumor formation ability using limiting dilution assay was performed in shSCR or shKDM5B-infected T-47D cells. The tumor volume was calculated, and the frequency of tumor formation was determined by ELDA. Quantitative analysis was performed using ImageJ software (version 1.50 g). Data were shown as mean ± SD. The data were analyzed by two-tailed unpaired *t*-test. *p < 0.05, **p < 0.01.

Given that KDM5B was significantly upregulated and correlated with the prognosis of patients with ER+ breast cancer, we investigated its function in ER+ breast cancer cells. To generate KDM5B knockdown lines, MCF-7 and T-47D cells were transfected with siRNAs targeting KDM5B (Fig. S3A, B). Consistent with previous findings [32], KDM5B knockdown decreased the proliferation of ER+ breast cancer cells (Fig. S3C, D). Additionally, stable KDM5B-knockdown or -overexpression breast cancer cell lines were generated via lentivirus infection (Fig. S3E, F). KDM5B overexpression enhanced the proliferation of ER+ breast cancer cells (Fig. 2A), whereas its knockdown significantly decreased the proportion of EdU-labeled cells and impaired their colony-forming ability (Figs. 2B and S3G). Transwell assays showed that overexpressed KDM5B enhanced, while shKDM5B attenuated, the invasive ability of ER+ breast cancer cells (Fig. 2C, D). Epithelial-mesenchymal transition (EMT), a hallmark of cancer, plays an important role in tumor metastasis [25, 33]. Therefore, we examined the expression of EMT markers in MCF-7 and T-47D cells following KDM5B knockdown or overexpression. KDM5B overexpression downregulated the expression of epithelial markers (E-cadherin, α-catenin, and γ-catenin) and upregulated the expression of mesenchymal markers (N-cadherin, Vimentin, and Fibronectin) (Fig. S3I).

RNAi screening in mice revealed that KDM5B is a major regulator of mouse hematopoietic stem cells (HSCs) [34, 35]. We examined the function of KDM5B in ER+ breast cancer cell stemness. KDM5B overexpression enhanced the sphere formation ability of ER+ breast cancer cells, whereas its knockdown significantly decreased the diameter of the spheres (Fig. 2E, F). Additionally, limiting dilution assay revealed that KDM5B overexpression improved the tumor formation frequency in mice transplanted with MCF-7 cells (Fig. 2G). In contrast, KDM5B knockdown markedly decreased the tumor volume and frequency of T-47D breast cancer cells (Fig. 2H). Moreover, KDM5B overexpression upregulated the protein expression of breast cancer stem cell markers, including OCT4, KLF4, SOX2, c-Myc, and NANOG (Fig. S3J). Collectively, these results demonstrate that KDM5B promotes the proliferation, invasion, and stemness of ER+ breast cancer cells.

### KDM5B interacts with the CRL4B complex

**Fig. 3:**
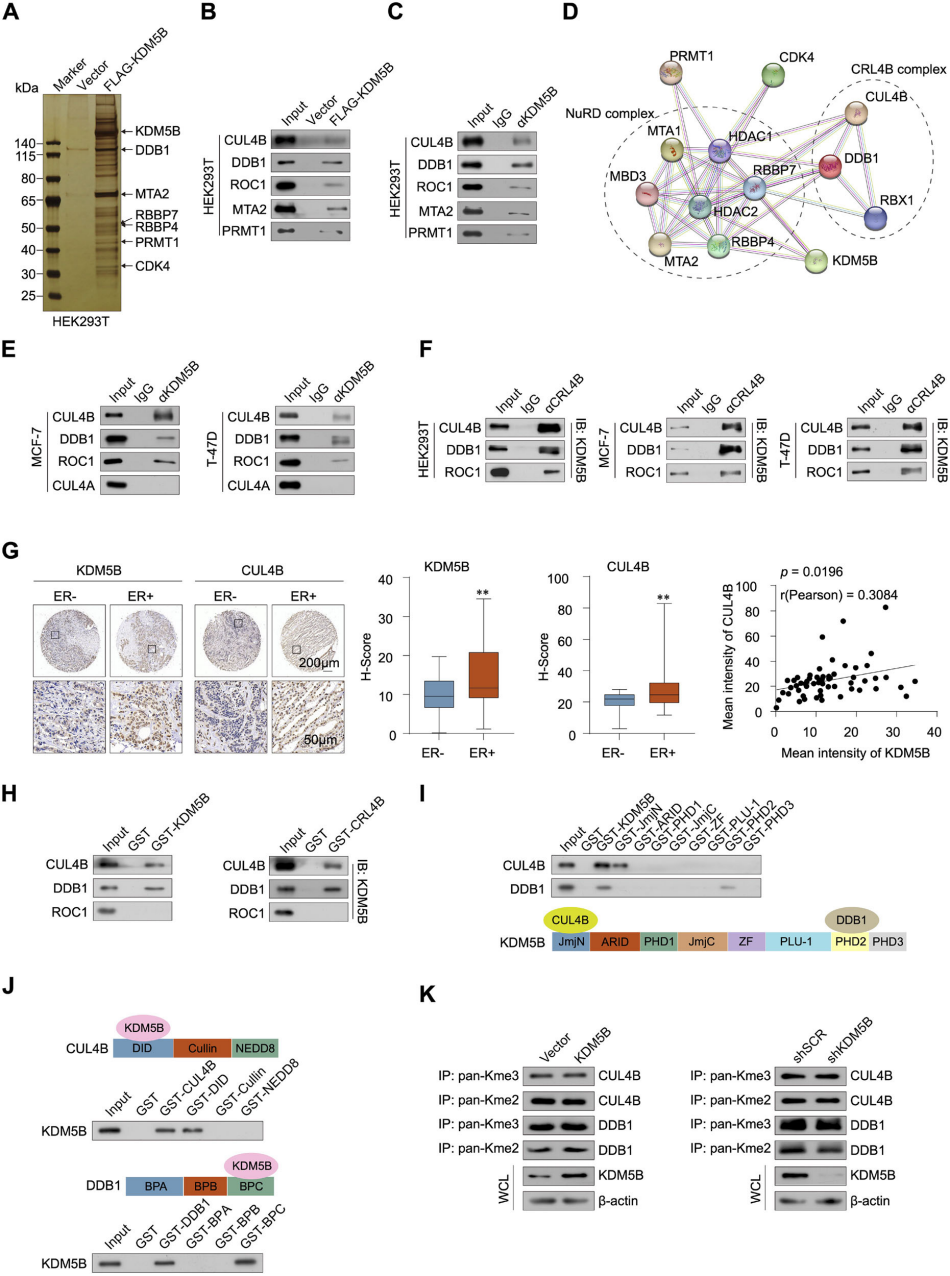
KDM5B physically associated with the CRL4B complex. **A** The HEK293T cells were transfected with FLAG-tagged KDM5B vector and IP-MS was performed to identify KDM5B-associated proteins. **B** Verifying the KDM5B-associated proteins in KDM5B-overexpressed HEK293T cells. **C** Verifying the KDM5B-associated proteins in HEK293T cells using KDM5B antibody. **D** The KDM5B-associated proteins were subjected to STRING website (https://string-db.org/) to reveal the association network. **E**, **F** The association between KDM5B and CRL4B complex was verified in MCF-7 and T-47D cells using IP experiments. **G** The expression of KDM5B and CUL4B was detected by IHC in breast cancer specimens. The IHC score was calculated using ImageJ software (version 1.5 g). **H** GST pulldown assays were performed to identify the physically associated proteins with KDM5B. **I**, **J** GST pulldown assays were performed to illustrate the molecular mechanisms between KDM5B and CRL4B complex. **K** Verifying KDM5B is not a demethylase for CUL4B and DDB1. Data were shown as mean ± SD. The data were analyzed by two-tailed unpaired t-test. **p < 0.01.

To identify KDM5B-interacting proteins, we performed IP-MS analysis of HEK293T cells overexpressing FLAG-tagged KDM5B (Fig. S4A). Notably, several proteins interacted with KDM5B, including DDB1, MTA2, and PRMT1 (Fig. 3A, B), which was confirmed in HEK293T cells by co-immunoprecipitating with anti-KDM5B antibody (Fig. 3C). MTA2 is a core component of the NuRD complex [36]. Although KDM5B has been proved to directly interact with the NuRD complex [37], there is no evidence of a direct interaction between KDM5B and the CRL4B complex (Fig. 3D). Given that DDB1 is also a component of the CRL4A complex [38], we performed IP assays to investigate the interaction between KDM5B and CRL4B or CRL4A in MCF-7 and T-47D cells. Notably, KDM5B interacted with the CRL4B complex but not with the CRL4A complex (Fig. 3E). To validate these interactions, antibodies against the CRL4B components, including CUL4B, DDB1, and ROC1, were used to detect the interactions with KDM5B. As shown in Fig. 3F, KDM5B was efficiently co-immunoprecipitated with the CRL4B complex. An analysis of the TCGA dataset revealed that KDM5B was significantly correlated with CUL4B and DDB1 in ER+ breast cancer (Fig. S4B). In the IHC chip of breast cancer tissues, KDM5B and CUL4B staining intensity was higher in ER+ samples than in ER- samples, and the expression levels of KDM5B and CUL4B were positively correlated (Fig. 3G).

To further explore the molecular basis of the interaction between KDM5B and the CRL4B complex, we conducted GST pull-down assays using GST-fused KDM5B or CRL4B complexes (Fig. S4C, D) and in vitro transcribed/translated CRL4B components or KDM5B, respectively. The results showed that KDM5B directly interacted with the CUL4B and DDB1, while ROC1 did not interact with KDM5B (Fig. 3H). To identify the motifs responsible for this interaction, we truncated KDM5B, CUL4B, and DDB1 into several GST-fused proteins (Fig. S4C, D). Notably, the JmjN and PHD2 domains of KDM5B were responsible for its interactions with CUL4B and DDB1, respectively (Fig. 3I). The DID domain of CUL4B and the C-terminal of DDB1 were responsible for the interaction with KDM5B (Fig. 3J). In addition, when there is no lysine methylation in ROC1 (Fig. S4E), the demethylation effect of KDM5B on CUL4B and DDB1 was negligible (Fig. 3K). Overall, these findings elucidate the molecular mechanisms underlying the interaction between KDM5B and the CRL4B complex.

### KDM5B/CRL4B complex promotes the proliferation, invasion, and stemness of ER+ breast cancer cells

**Fig. 4:**
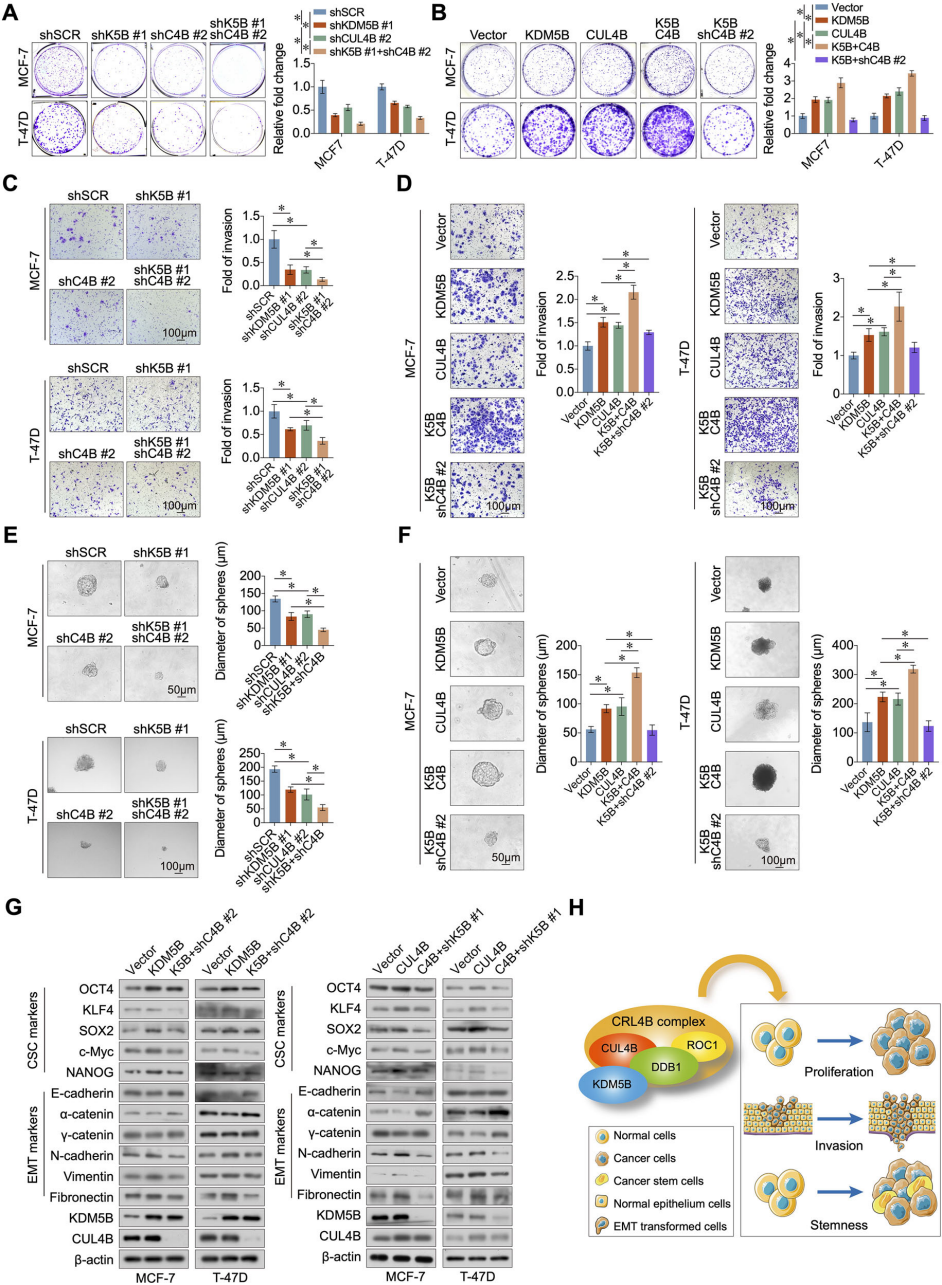
KDM5B, together with the CRL4B complex, promoted the proliferation, invasion, and stemness of ER+ breast cancer cells. **A** Colony formation assay was conducted to evaluate the growth ability of KDM5B-deficient MCF-7 and T-47D cells acquiring CUL4B knockdown simultaneously. **B** Colony formation assay was conducted to evaluate the growth ability of KDM5B-overexpressed MCF-7 and T-47D cells acquiring CUL4B overexpression or knockdown simultaneously. **C** Transwell experiment was conducted to evaluate the invasive ability of KDM5B-deficient MCF-7 and T-47D cells acquiring CUL4B knockdown simultaneously. **D** Transwell experiment was conducted to evaluate the invasive ability of KDM5B-overexpressed MCF-7 and T-47D cells acquiring CUL4B overexpression or knockdown simultaneously. **E** Sphere formation assay was conducted to evaluate the stemness of KDM5B-deficient MCF-7 and T-47D cells acquiring CUL4B knockdown simultaneously. **F** Sphere formation assay was conducted to evaluate the stemness of KDM5B-overexpressed MCF-7 and T-47D cells acquiring CUL4B overexpression or knockdown simultaneously. **G** Western blot was performed to detect the EMT and CSC markers in KDM5B- or CUL4B-overexpressed MCF-7 and T-47D cells with or without CUL4B- or KDM5B-knockdown, respectively. β-actin was used as an internal control. **H** Graphic model illustrated that KDM5B cooperated with the CRL4B complex through physically interacting with CUL4B and DDB1 to promote the proliferation, invasion, and stemness of ER+ breast cancer cells. Quantitative analysis was completed with ImageJ software (version 1.5 g). Data were shown as mean ± SD. The data were analyzed by One-way ANOVA. *p < 0.05. shK5B shKDM5B, shC4B shCUL4B, K5B KDM5B, C4B CUL4B.

Based on our results, we investigated whether the CRL4B complex was involved in the KDM5B-mediated malignant phenotypes of breast cancer cells by overexpressing or knocking down CUL4B in MCF-7 and T-47D cells (Fig. S5A, B). CUL4B knockdown decreased the proliferation of MCF-7 and T-47D cells (Fig. 4A), whereas its overexpression had the opposite effect (Fig. 4B). Double knockdown of KDM5B and CUL4B further suppressed cell proliferation (Fig. 4A). In contrast, CUL4B overexpression enhanced the proliferation of ER+ breast cancer cells overexpressing KDM5B (Fig. 4B). CUL4B knockdown or overexpression significantly decreased or enhanced the invasion abilities of MCF-7 and T-47D cells with or without KDM5B knockdown (Fig. 4C, D). We previously found that KDM5B contributed the stemness of ER+ breast cancer cells (Fig. 2E–H). To evaluate the role of CUL4B in the stemness of breast cancer cells, we conducted sphere formation assays using MCF-7 and T-47D cells. CUL4B knockdown decreased the diameter of spheres generated by these two ER+ breast cancer cells with or without KDM5B knockdownt (Fig. 4E), whereas CUL4B overexpression promoted sphere formation (Fig. 4F). Similarly, CUL4B knockdown significantly decreased the diameter of spheres induced by KDM5B overexpression (Fig. 4F). To validate these scenarios, we examined the protein levels of CSC- and EMT-associated markers in MCF-7 and T-47D cells. KDM5B or CUL4B overexpression upregulated the expression of CSC markers (OCT4, KLF4, SOX2, c-Myc, and NANOG) and mesenchymal markers (N-cadherin, vimentin, and fibronectin) and downregulated the expression of epithelial markers (E-cadherin, α-catenin, and γ-catenin) (Fig. 4G). In contrast, simultaneous knockdown of CUL4B or KDM5B reversed the expression of these proteins (Fig. 4G). Collectively, these results demonstrate that KDM5B forms a complex with CRL4B to promote the proliferation, invasion, and stemness of ER+ breast cancer cells (Fig. 4H).

### Identifying the genome-wide transcription targets of the KDM5B/CRL4B complex

**Fig. 5:**
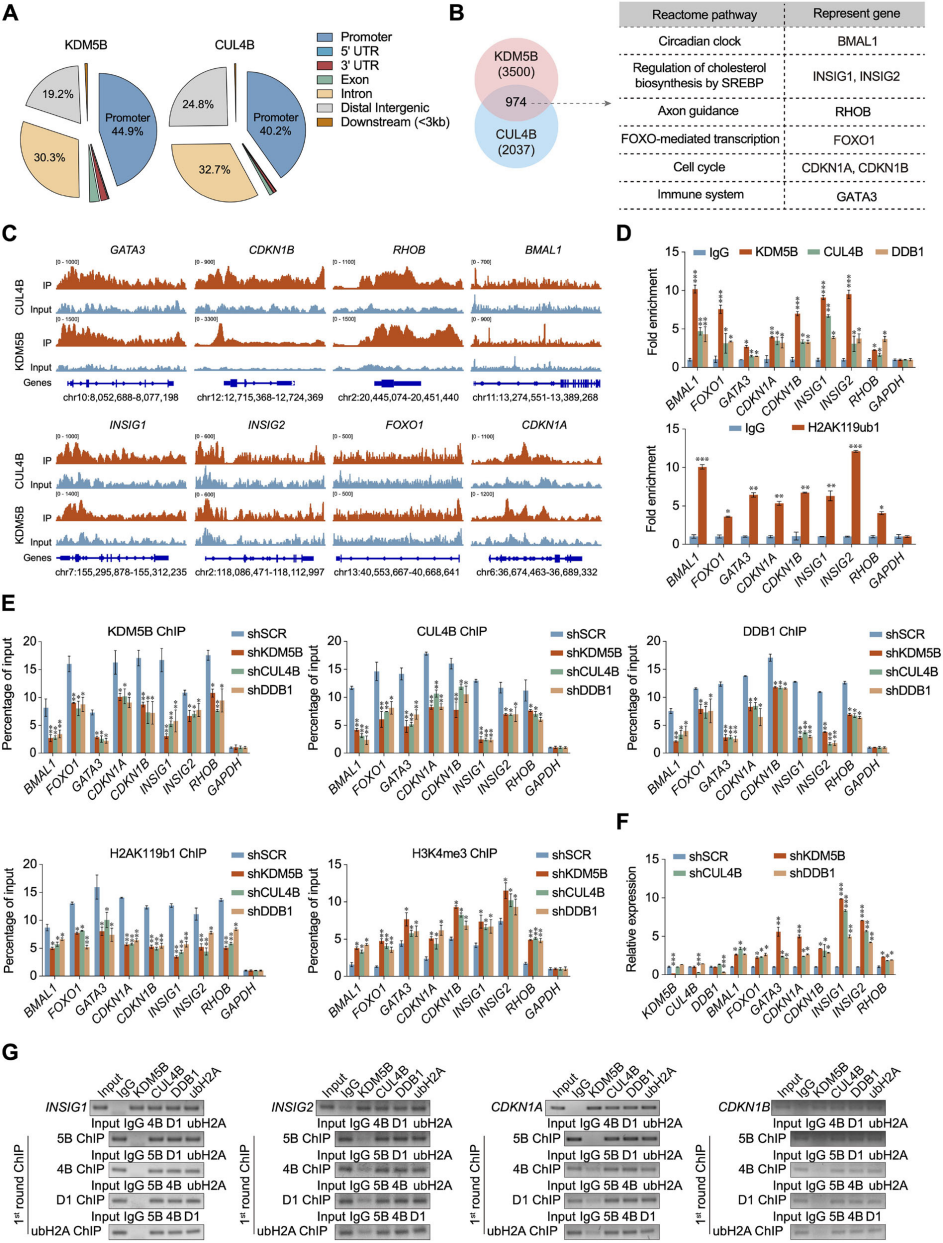
Identification of genome-wide transcription targets of the KDM5B/CRL4B complex. **A** Genomic distribution of KDM5B and CUL4B determined by ChIP-seq analysis in MCF-7 cells. **B** Left panel, Venn diagram of overlapping promoters bound by KDM5B and CUL4B in MCF-7 cells. Numbers represent the number of promoters targeted by the indicated proteins. Right panel, Representative genes of 6 enriched reactome pathways were shown. **C** Binding profiles of KDM5B and CUL4B on the representative target genes GATA3, CDKN1A, CDKN1B, RHOB, BMAL1, INSIG1, INSIG2, and FOXO1. **D** Verification of ChIP-seq results using ChIP-qPCR analysis of target genes identified in MCF-7 cells. Results are represented as fold change over control. GAPDH served as the internal control. **E** Verification of ChIP-seq results using ChIP-qPCR analysis of target genes identified in MCF-7 cells with KDM5B-, CUL4B- or DDB1-knockdown. GAPDH served as the internal control. **F** mRNA levels of indicated genes in the KDM5B-, CUL4B-, and DDB1- knockdown MCF-7 cells. **G** ChIP and Re-ChIP analysis in MCF-7 cells with indicated antibodies. 5B, KDM5B; 4B, CUL4B; D1, DDB1; K119ub1, H2AK119ub1. Data were shown as mean ± SD. The data were analyzed by two-tailed unpaired *t*-test or One-way ANOVA. *p < 0.05, **p < 0.01, ***p < 0.001.

To elucidate the mechanisms of the KDM5B/CRL4B complex, we performed ChIP-seq using antibodies targeting KDM5B and CUL4B in MCF-7 cells. After calling peaks, we obtained a similar distribution of KDM5B and CUL4B in different gene regions, including the promoter, exon, and intron (Fig. 5A), with the highest enrichment of these two proteins in the gene promoters. Importantly, these genes were involved in multiple signaling pathways, including the circadian clock, cholesterol biosynthesis, axon guidance, FOXO-mediated transcription, cell cycle, and immune system (Fig. 5B). Additionally, we selected eight representative genes within these pathways to validate our ChIP-seq results (Fig. 5B, C). Moreover, we performed the ChIP-qPCR experiments to detect the enrichment of the KDM5B/CRL4B complex in the promoters of the selected genes. Consistent with the sequencing results, KDM5B and the components of the CRL4B complex, CUL4B and DDB1, were significantly enriched in the promoter regions (Fig. 5D). We investigated the enrichment of H2AK119ub1 in these promoter regions and the results confirmed this proposal (Fig. 5D). Our study revealed that KDM5B physically interacts with the CRL4B complex and co-regulates downstream target genes. To investigate whether knocking down any of the components affected the function of the KDM5B/CRL4B complex, we evaluated the enrichment of each component in the promoter regions in MCF-7 cells following KDM5B, CUL4B or DDB1 knockdown. Expectedly, there was a decrease in the enrichment of KDM5B, CUL4B, DDB1, and H2AK119ub1 at the promoters in MCF-7 cells (Fig. 5E). KDM5B is a histone demethylation enzyme that catalyzes the demethylation of histone H3K4me3, a transcriptional activation marker (Fig. S5C). KDM5B, CUL4B or DDB1 knockdown in MCF-7 cells increased the enrichment of H3K4me3 marker in the promoters of the target genes, demonstrating transcriptional activation of these genes (Fig. 5E). Additionally, KDM5B, CUL4B, and DDB1 knockdown upregulated the mRNA levels of these genes in MCF-7 cells (Fig. 5F). To further verify the hypothesis that KDM5B coordinates with the CRL4B complex to regulate unique target genes, we performed ChIP/reChIP assays to detect the enrichment of these proteins or epigenetic markers in the promoters of INSIG1/2 and CDKN1A/B genes. Soluble chromatin was immunoprecipitated with antibodies targeting KDM5B, CUL4B, DDB1, and H2AK119ub1 (Fig. 5G), indicating that these genes are co-regulated by the KDM5B/CRL4B complex. Additionally, the first-round ChIP elutes were re-immunoprecipitated with the indicated antibodies, and the results showed that the representative promoters were eluted and amplified via PCR (Fig. 5G). Collectively, these results reveal that KDM5B coordinates with the CRL4B complex to suppress the transcription of downstream target genes by monoubiquitinating H2AK119 and demethylating trimethylated H3K4 in breast cancer cells.

### KDM5B/CRL4B complex promotes the invasion of breast cancer cells by regulating cholesterol biosynthesis

**Fig. 6:**
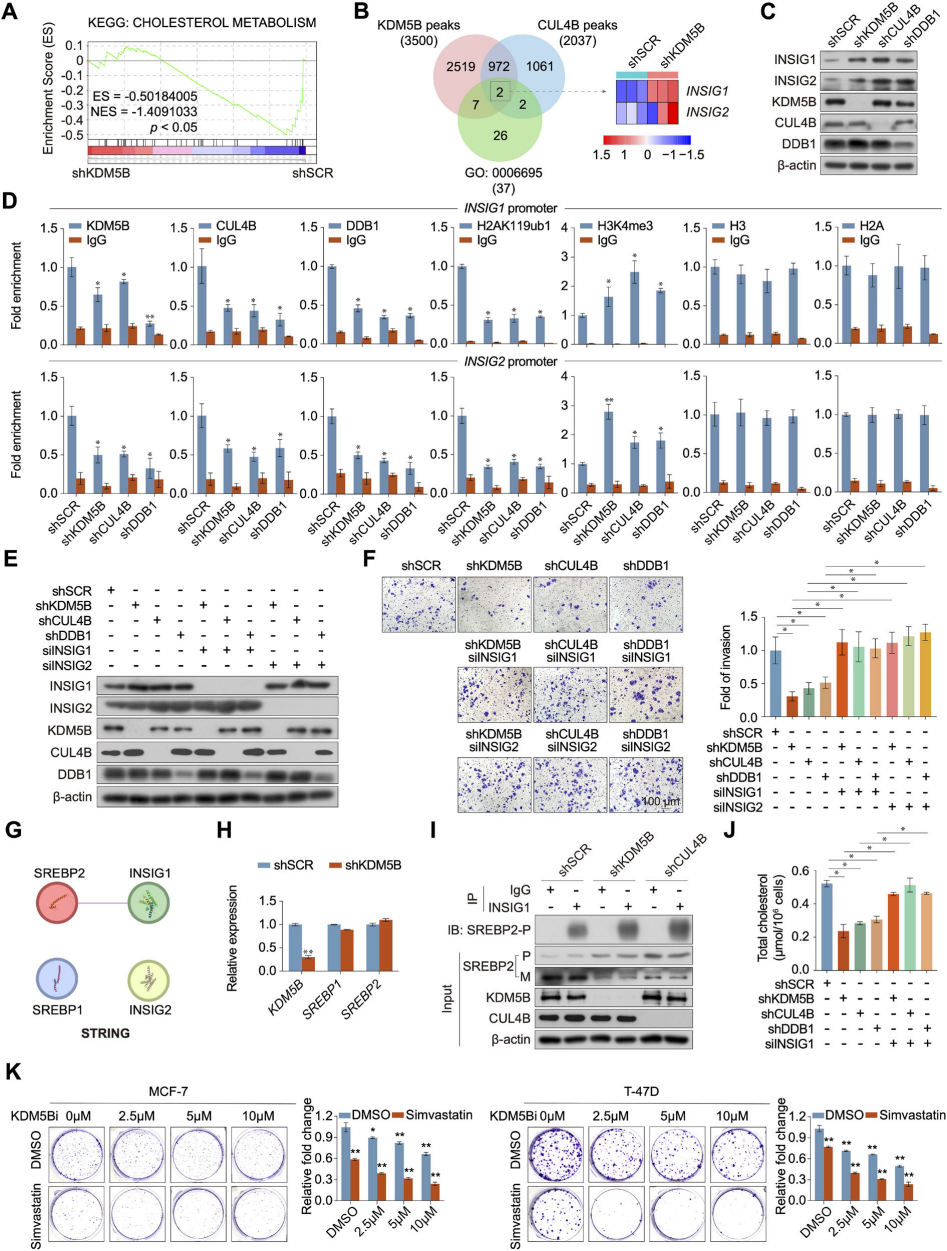
KDM5B promoted the invasion of MCF-7 cells through regulating cholesterol metabolism. **A** The cholesterol metabolism pathway was regulated by KDM5B in MCF-7 cells using GSEA analysis of RNA-seq results. **B** Venn diagram of overlapping genes involved in cholesterol biosynthesis (GO: 0006695) and regulated by KDM5B and CUL4B in MCF-7 cells. **C** Protein levels of INSIG1 and INSIG2 were detected by western blot in KDM5B-, CUL4B- or DDB1-depleted MCF-7 cells. β-actin was used as an internal control. **D** Recruitment of indicated proteins to INSIG1 and INSIG2 promoters in MCF-7 cells after transfection with control shSCR or shRNAs targeting KDM5B, CUL4B or DDB1. Purified rabbit IgG was used as a negative control. **E** INSIG1 and INSIG2 levels were detected by western blot in KDM5B-, CUL4B- or DDB1-deficient MCF-7 cells with or without transfection of siRNAs targeting INSIG1 or INSIG2. β-actin was used as an internal control. **F** The invasive ability was evaluated by transwell experiment in KDM5B-, CUL4B- or DDB1-deficient MCF-7 cells with or without transfection of siRNAs targeting INSIG1 or INSIG2. **G** The interactions of SREBPs and INSIGs were predicted using STRING website. **H** mRNA levels of SREBP1 and SREBP2 were evaluated in MCF-7 cells acquiring KDM5B-knockdown. **I** The co-IP experiment was conducted using INSIG1 antibody for immunoprecipitating SREBP2-precusor in MCF-7 cells transfected with shSCR or shRNAs targeting KDM5B or CUL4B. Purified rabbit IgG was used as a negative control. β-actin was used as an input control. **J** The total cholesterol levels were determined in KDM5B-, CUL4B- or DDB1-deficient MCF-7 cells with or without INSIG1 knockdown. **K** The proliferation status of breast cancer cells under simvastatin and KDM5B inhibitor treatment. Data were shown as mean ± SD. The data were analyzed by two-tailed unpaired *t*-test or One-way ANOVA. *p < 0.05, **p < 0.01.

In normal cells, cholesterol biosynthesis is regulated by the sterol regulatory element-binding protein 2 (SREBP2) to sustain the cholesterol homeostasis (Fig. S6A). Our study revealed that KDM5B promoted the tumorigenesis of ER+ breast cancer cells by regulating multiple signaling pathways, including cholesterol metabolism (Fig. 6A). Moreover, the regulatory effect of KDM5B on cholesterol levels was more pronounced in ER+ than in ER- breast cancer cells (Fig. S6B). Accordingly, we re-analyzed our sequencing data and found that the cholesterol-inhibitory molecules INSIG1/2, involved in the cholesterol biosynthetic pathway, were transcriptionally regulated by the KDM5B/CRL4B complex (Fig. 6B). Additionally, silencing any one of the complex components (KDM5B, CUL4B, or DDB1) significantly upregulated INSIG1 and INSIG2 protein levels in MCF-7 cells (Fig. 6C). Moreover, disrupting the complex decreased the enrichment of the KDM5B/CRL4B complex in the promoter regions of INSIG1 and INSIG2 in MCF-7 cells, downregulated the corresponding histone monoubiquitination at H2AK119 and increased the histone activation marker H3K4 trimethylation in the INSIG1 and INSIG2 promoters (Fig. 6D). To reveal the role of INSIG1 and INSIG2 in KDM5B/CRL4B-mediated malignant phenotype in ER+ breast cancer cells, we transiently downregulated INSIG1 or INSIG2 expression using siRNAs in KDM5B/CRL4B-disrupted MCF-7 cells (Fig. 6E). INSIG1 or INSIG2 knockdown enhanced the invasive capacity of KDM5B, CUL4B, or DDB1-depleted MCF-7 cells (Fig. 6F).

INSIG1/2 proteins serve as brakes to balance cholesterol levels in mammalian cells (Fig. S6A). However, INSIG1 was shown to interact with SREBP2 according to the STRING public database (Fig. 6G). To investigate whether KDM5B regulates SREBP2 maturation, we examined the transcription correlation between KDM5B and SREBP2. Importantly, KDM5B knockdown did not affect the transcription levels of SREBP1 or SREBP2 (Fig. 6H). In contrast, KDM5B or CUL4B knockdown significantly enhanced the interaction between INSIG1 and SREBP2 precursor by inhibiting the cleavage of SREBP2 (Fig. 6I). Consequently, disrupting the KDM5B/CRL4B complex decreased the total cholesterol levels in MCF-7 cells, whereas INSIG1 knockdown reversed this effect (Fig. 6J). Notably, treatment with the cholesterol inhibitor simvastatin significantly decreased the growth rate of breast cancer cells (Fig. S6C) and downregulated the expression of genes related to the cholesterol synthesis pathway (Fig. S6D). Additionally, the combination of simvastatin significantly enhanced the inhibitory effect of the KDM5B inhibitor on breast cancer cell proliferation (Fig. 6K). Overall, these findings demonstrate that the KDM5B/CRL4B complex promotes the invasion of ER+ breast cancer cells by regulating cholesterol biosynthesis via downregulating INSIG1 and INSIG2 expression.

### KDM5B expression is regulated by 27-hydroxycholesterol (27-HC) in ER+ breast cancer cells

**Fig. 7:**
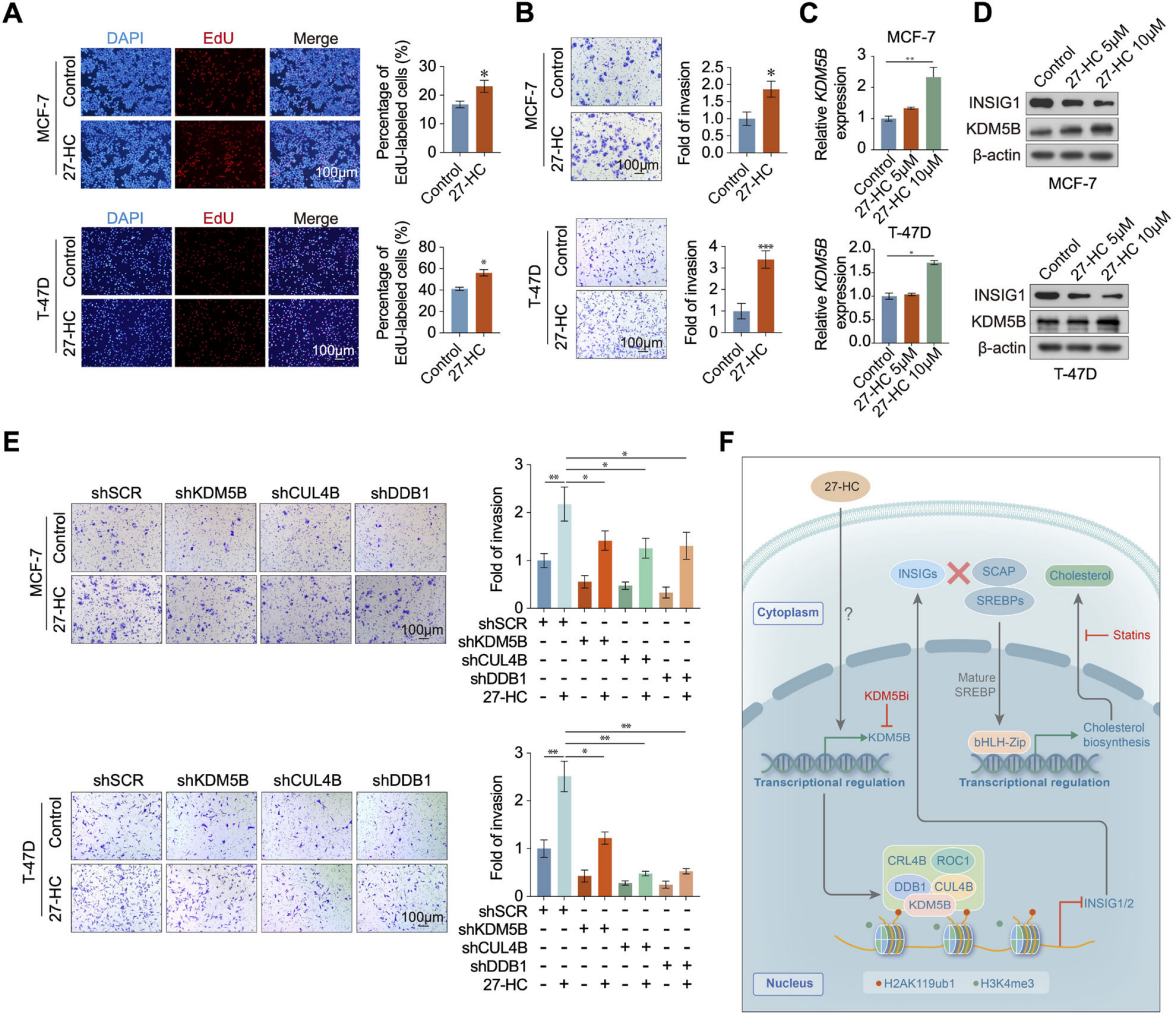
KDM5B expression was regulated by 27-HC in ER+ breast cancer cells. **A** EdU assays were used to investigate the proliferation of MCF-7 cells under 10 μM 27-HC treatment. **B** Transwell experiments were used to investigate the invasion of MCF-7 cells under 10 μM 27-HC treatment. **C**, **D** The transcription and protein levels were detected by qPCR and western blot in MCF-7 and T-47D cells with 27-HC treatment. **E** The invasive ability was evaluated in KDM5B-, CUL4B- or DDB1-deficient MCF-7 cells with or without 27-HC treatment. **F** Graphic model as discussed in the text. The proposed regulatory mechanisms of the KDM5B/CRL4B complex in ER+ breast carcinogenesis through regulating the cholesterol metabolism. Data were shown as mean ± SD. The data were analyzed by two-tailed unpaired *t*-test or One-way ANOVA. *p < 0.05, **p < 0.01, ***p < 0.001.

27-HC is the most well-studied oxysterol and is elevated to promote proliferation and tumor growth in ER+ breast cancer [28]. Among several cholesterol metabolites, 27-HC is the most abundant oxysterol that increases the risk of breast cancer progression [39]. We confirmed that 27-HC promoted the proliferation and invasion of MCF-7 and T-47D cells (Fig. 7A, B). Treatment with 27-HC upregulated KDM5B expression in ER+ breast cancer cells at both the mRNA and protein levels (Fig. 7C, D) and downregulated INSIG1 and INSIG2 expression in MCF-7 and T-47D cells (Fig. 7D). To investigate the role of 27-HC in KDM5B/CRL4B-mediated increase in the invasion capacity of ER+ breast cancer cells, we conducted transwell assays in KDM5B, CUL4B, or DDB1-depleted cells treated with 27-HC. Treatment with 27-HC significantly weakened the invasive ability of breast cancer cells with disrupted KDM5B/CRL4B complex (Fig. 7E), indicating that 27-HC promotes the proliferation and invasion of ER+ breast cancer cells, partly by upregulating KDM5B expression.

Our study revealed upstream and downstream regulations of KDM5B in ER+ breast cancer cells. 27-HC treatment upregulated KDM5B expression via unknown mechanisms, and KDM5B coordinated with the CRL4B complex to inhibit INSIG1 and INSIG2 expression, promoting the proliferation, invasion, and stemness of ER+ breast cancer cells by regulating cholesterol metabolism (Fig. 7F).

### KDM5B expression is upregulated and associated with the prognosis of patients with multiple cancer types

**Fig. 8:**
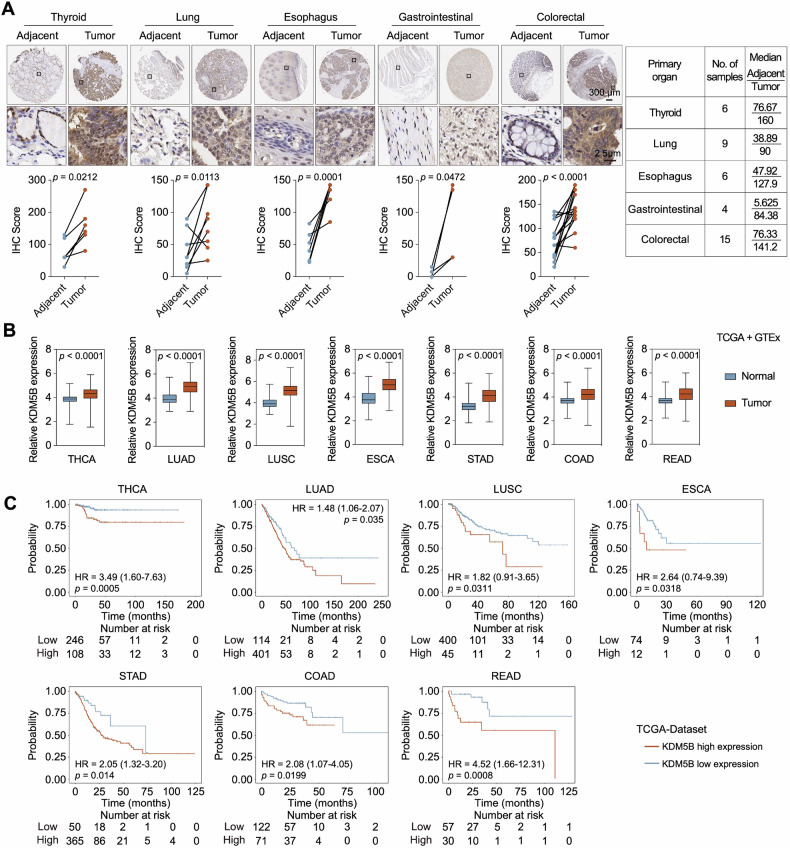
KDM5B was upregulated and predicted poor prognosis in multiple cancers. **A** The expression of KDM5B was detected by IHC in various human cancer specimen and adjacent normal tissues. The IHC score was calculated using ImageJ software (version 1.5 g). **B** The TCGA datasets were used to evaluate the KDM5B expression in multiple cancer types. **C** The correlation of KDM5B expression and survival probability was analyzed using TCGA datasets. Data were shown as mean ± SD. The data were analyzed by two-tailed unpaired *t*-test.

To investigate the role of KDM5B in other cancer types, we examined its expression in tumor specimens and adjacent normal tissues obtained from patients with different types of cancers using immunohistochemistry (IHC). KDM5B expression was upregulated in multiple tumors, including thyroid, lung, esophageal, gastrointestinal, and colorectal cancers (Fig. 8A). To confirm this, we analyzed the TCGA and GTEx datasets and confirmed that KDM5B expression was elevated in these cancers (Fig. 8B). Additionally, the TCGA data revealed that high KDM5B expression was correlated with a low survival rate in these cancer types (Fig. 8C). Overall, these data demonstrate that KDM5B is upregulated in multiple carcinomas and is a potential therapeutic target.

## Discussion

KDM5B, a luminal-driven oncogene in breast cancer, is positively correlated with transcriptomic heterogeneity in ER+ but not in ER- breast tumors [13, 40]. Our study showed that KDM5B was upregulated and positively correlated with poor prognosis in breast cancer, especially in ER+ breast tumors. KDM5B overexpression promotes the proliferation and invasion of ER+ breast cancer cells. Consistent with previous findings [34, 35, 41, 42], KDM5B expression was strongly associated with the stemness of ER+ breast cancer cells. Contrary to the findings in ER+ breast cancer cells, KDM5B expression was downregulated in triple-negative breast cancer (TNBC) cells, such as MDA-MB-231 and Hs 578T cells. Moreover, KDM5B overexpression in triple-negative breast cancer cells suppresses cell migration and invasion, making KDM5B a potential tumor suppressor in TNBC [18]. However, the detailed mechanisms and regulatory networks underlying the role of KDM5B remain unclear. Although KDM5B is upregulated in other cancers, such as hepatocellular carcinoma and prostate cancer [43, 44], its expression is inhibited by the PRC2 complex, leading to acute myeloid leukemia (AML) growth [37]. TCGA dataset analysis showed that KDM5B expression was upregulated in several cancer types, including lung, colorectal, and gastric cancers, suggesting that KDM5B may be a promising therapeutic target in ER+ breast cancer.

KDM5B belongs to the JmjC domain-containing histone demethylases that have dual functions in gene transcriptional activation and repression, promoting or inhibiting tumor growth [45, 46]. As a histone H3K4me2/3 lysine demethylase, KDM5B regulates the transcription of several target genes involved in multiple signaling pathways, including the immune system [47, 48], cell cycle [44], and PI3K-AKT signaling [49]. Our findings showed that KDM5B is involved in several other pathways, including the circadian rhythm and cholesterol metabolism. Our previous report revealed that the PRMT6/PARP1/CRL4B complex disrupts the circadian clock to promote breast cancer development by regulating core clock genes such as PER3 [24]. In the present study, IP assays showed that KDM5B physically interacted with the CRL4B complex, resulting in elevated proliferation, invasion, and stemness of ER+ breast cancer cells. Additionally, we found that the core clock gene BMAL1 can be regulated by the KDM5B/CRL4B complex. However, the regulatory networks and underlying mechanisms require further investigation. KDM5B coordinates with the NuRD complex in breast cancer cells and functions as a tumor suppressor in TNBC cells, suggesting that KDM5B may be a major regulator that exerts distinct functions in different subtypes of breast cancer [18]. In this research, the authors discovered that the PHD1 and PHD3 domains of KDM5B (but not PHD2 domain) exhibited histone-binding activity. Additionally, the PHD1 domain recognizes unmodified or methylated H3K9, whereas the PHD3 domain shows a preference for H3K4me3. In the present study, KDM5B interacted with the C-terminus of DDB1 through the PHD2 domain, demonstrating that the PHD2 domain is a protein-binding motif of KDM5B. Unlike other Jumonji-containing histone demethylases, the KDM5 family is unique because the Jumonji domain is separated into JmjN and JmjC domains by the insertion of ARID-PHD1 domain [50]. Histone demethylase inhibitors, such as GSK-J4 and JIB-04, induce antiproliferative effects in breast cancer cells [50]. In the present study, the JmjN domain directly interacted with the N-terminus of CUL4B to promote breast tumorigenesis. Speculatively, the JIB-04 may target the interaction between KDM5B and CUL4B to disrupt the formation of the complex, thereby inhibiting the growth of MCF-7 cells. However, further research is required to validate this hypothesis.

Fast-proliferating cells, such as cancer cells, require high levels of cholesterol for membrane biogenesis and other functions [51]. In breast cancer, the oncometabolite 6-oxo-cholestan-3β,5α-diol (OCDO), derived from cholesterol, is enriched in patients with breast cancer and promotes tumor progression [52]. Increased cholesterol biosynthesis is a hallmark of several cancers. Moreover, the central regulator of cholesterol biosynthesis, SREBP2, is regulated by the NUCB2/Nesfatin-1 axis to promote the metastasis of breast cancer cells [53]. Therefore, inhibiting the transcriptional activation of SREBP2 is a promising strategy for cancer therapy. To the best of our knowledge, this study is the first to show that the KDM5B/CRL4B complex promotes cholesterol biosynthesis in ER+ breast cancer by downregulating the SREBP2-binding brakes INSIG1/2. Our observations suggest that agents capable of reactivating INSIG1/2 transcription in breast tumor cells may be effective for treating breast cancers.

27-HC, the most well-studied cholesterol metabolite, has been shown to contribute to the growth and metastasis of several cancers, especially ER+ breast cancer. Moreover, previous studies have confirmed that 27-HC is an endogenous SERM [54, 55]. Our study showed that 27-HC regulates KDM5B expression in ER+ breast cancer cells. Additionally, some findings suggest that 27-HC acts as an LXR ligand [56]. Therefore, 27-HC may regulate KDM5B through the ERα or LXR pathway. Additionally, the LXR pathway regulates cholesterol efflux, and its activation inhibits the proliferation of ER+ breast cancer cells [57]. 27-HC is a primary metabolite of cholesterol and is catalyzed by CYP27A1 enzyme, making it a druggable target [58]. Inhibition of CYP27A1 significantly reduced tumor cell metastasis in mouse models [59]. Additionally, 27-HC treatment decreased the number of cytotoxic CD8 + T cells (CTLs) [59], indicating that KDM5B may play a role in remodeling the tumor microenvironment. Additionally, KDM5B promotes immune evasion through the cGAS-STING pathway, leading to suppressed anti-tumor immunity [47]. This is a promising research direction for unlocking the immune regulatory role of KDM5B in breast cancer.

Despite these promising findings, this study had some limitations. For example, it is unclear whether KDM5B regulates 27-HC expression. Additionally, the specific mechanisms by which 27-HC regulates KDM5B expression remain unclear. Moreover, the effects of 27-HC downregulation on the therapeutic efficiency of KDM5B inhibitors require further investigation.

In conclusion, our study confirmed that KDM5B is an oncogene in breast cancer, particularly in ER+ breast cancer. KDM5B expression is elevated in breast cancer and other types of cancer, including lung, colon and gastric cancers. KDM5B contributes to the proliferation, invasion, and stemness of breast cancer cells by regulating cholesterol metabolism. Mechanistically, KDM5B physically interacts with the CRL4B complex to inhibit the transcriptional expression of INSIG1 and INSIG2 by modifying H2AK119 ubiquitination and H3K4 trimethylation levels. Moreover, the cholesterol metabolite 27-HC is an upstream regulator of KDM5B, leading to malignant phenotypes.

## Materials and methods

### Reagents and antibodies

Anti-KDM5B antibody for IHC, anti-CUL4B, anti-vimentin, anti-β-actin, and anti-FLAG antibodies were purchased from Sigma-Aldrich (Burlington, MA, USA). Anti-KDM5B antibodies for immunoblotting and immunoprecipitation, anti-fibronectin, anti-c-Myc, anti-SOX2, anti-OCT4, anti-ROC1, anti-MTA2, anti-PRMT1, anti-CUL4A, and anti-SREBP2 antibodies were purchased from Abcam (Cambridge, UK). The anti-KDM5B antibody for chromatin immunoprecipitation was purchased from NOVUS Biologicals (Littleton, CO, USA). Anti-E-cadherin, anti-α-catenin, anti-γ-catenin, and anti-N-cadherin antibodies were purchased from BD Biosciences (Franklin Lakes, NJ, USA). Anti-INSIG2 and anti-SREBP1 antibodies were purchased from Proteintech (Rosemont, IL, USA). Anti-INSIG1 antibody was purchased from Santa Cruz Biotechnology (Dallas, TX, USA) and anti-H3K4me3 antibody for immunoblotting was purchased from ABclonal (Wuhan, Hubei, China). Anti-KLF4, anti-NANOG, and anti- H2AK119ub1 antibodies were purchased from Cell Signaling Technology (Danvers, MA, USA). TransScript® One-Step gDNA Removal and cDNA Synthesis SuperMix and PerfectStart® Green qPCR SuperMix were purchased from TransGen (Beijing, China). Crystal violet staining solution was purchased from Beyotime (Shanghai, China). Cell-Light EdU Apollo®567 In Vitro Kit was purchased from RiboBio (Guangzhou, Guangdong, China). Dimethyl sulfoxide (DMSO) was obtained from VWR (Radnor, PA, USA). 27-HC was purchased from MCE (New Jersey, USA). EDTA, penicillin/streptomycin, TRIzol reagent, polyethylenimine (PEI), Lipofectamine RNAiMAX, and Opti-MEM were purchased from Thermo Fisher Scientific (Waltham, MA, USA). SYBR GREEN was purchased from Roche (Basel, Switzerland). High-glucose Dulbecco’s modified Eagle’s medium (DMEM) and fetal bovine serum (FBS) were purchased from Biological Industries (Sartorius, Gottingen, Germany). T7 Quick-coupled transcription/translation system was purchased from Promega (Madison, WI, USA). NucleoSpin Gel and PCR Clean-up Kits were purchased from MACHEREY-NAGEI (Düren, North Rhlne-Westphalia, Germany). Protease inhibitor cocktail was obtained from Roche Applied Science (Penzberg, Germany). All lentiviruses used in this study to generate knockdown or overexpressing cell lines were purchased from Genechem (Shanghai, China).

### Cell culture and transfection

HEK293T cells and breast cancer cells (MCF-7, T-47D, MDA-MB-231, Hs 578T, HCC1937, and MDA-MB-468) were obtained from the American Type Culture Collection (ATCC, Manassas, VA, USA). All cells were incubated in complete DMEM supplemented with 10% FBS at 37 °C in a humidified incubator under a 5% CO_2_ atmosphere. Transfections were performed using TurboFect Transfection Reagent (Thermo Fisher Scientific, Waltham, MA) and Lipofectamine RNAiMAX Reagent (Invitrogen, Carlsbad, CA) according to the manufacturer’s instructions. Each experiment was performed in triplicate. For RNAi experiments, at least three independent siRNA/shRNA sequences were tested for each gene and the one with the highest efficiency was used. Subsequent experiments were performed using the transfected cells. The shRNAs and siRNAs sequences used are listed in Table S1. The sequences of plasmids used in this study are listed in Table S2.

### Quantitative real-time PCR (RT-qPCR)

Total RNA was extracted from the cells using TRIzol reagent and reverse-transcribed to generate first-strand cDNA using TransScript® One-Step gDNA Removal and cDNA Synthesis SuperMix according to the manufacturer’s instructions. qPCR was performed on an ABI 7500 FAST detection System (Waltham, MA, USA) using SYBR GREEN. Finally, the relative expression of the target genes was calculated using the comparative Ct method, with β-actin as the internal control. This assay was performed in triplicate. Primers used for RT-qPCR are listed in Table S3.

### Immunoblotting and co-immunoprecipitation (co-IP)

Briefly, the cells were lysed using RIPA lysis buffer (Applygen, Beijing, China) containing protease inhibitor cocktail and centrifuged at 12,000 × *g* for 15 min at 4 °C. Thereafter, the supernatant was mixed with 5× SDS-PAGE loading buffer and boiling for 10 min. Proteins were separated using 10% SDS-PAGE and transferred to polyvinylidene fluoride membranes. After blocking with 5% nonfat milk, the membranes were incubated with specific primary antibodies overnight at 4 °C and further incubated with secondary antibodies. Protein bands were developed using an enhanced chemiluminescence system (Thermo Fisher Scientific). For the co-IP experiment, cells were collected and treated with lysis buffer (150 mM NaCl, 0.5% NP-40, 50 mM Tris-HCl, 1 mM EDTA, 0.25% sodium deoxycholate, and protease inhibitor) at 4 °C for 1 h. After centrifugation, the supernatant was collected and incubated with specific primary antibodies overnight at 4 °C under constant rotation. Thereafter, Dynabeads™ Protein G (Thermo Fisher) was added to the mixture and incubated for 2 h incubation. After washing, immunoblotting was performed as previously described.

### Proliferation assays

5-ethynyl-2′-deoxyuridine (EdU) and colony formation assays were performed to evaluate cell proliferation. EdU assay was performed using Cell-Light EdU Apollo567® In Vitro Kit according to the manufacturers’ instructions, and images were captured using a fluorescent microscope. For colony formation assay, 5000 cells were seeded in 6-well plate and cultured for 10 days at 37 °C. Thereafter, the cells were stained with crystal violet and images were captured.

### Invasion assay

Cell invasion capacity was evaluated with transwell assay. Briefly, cells (5 × 10^4^) suspended in 500 μl serum-free DMEM were added to the upper chamber of transwell inserts pre-coated with Matrigel Matrix (BD Biosciences). DMEM supplemented with 10% FBS (500 μl) was added to the lower chamber. Thereafter, the cells were cultured for 24 h at 37 °C, stained with crystal violet, and counted. Five high-powered fields were counted per membrane.

### Sphere formation assay

Sphere-formation assay was performed to assess cell stemness. Briefly, 5000 cells were seeded in 6-well ultralow-attachment plates in serum-free DMEM/F12 supplemented with 0.4% BSA, 20 ng/mL bFGF, 10 ng/mL EGF, and 5 µg/mL insulin. Fresh medium was added every 3 days for 2 weeks. Tumor spheres were counted and photographed using a microscope.

### Measurement of total cholesterol levels

Total cholesterol levels in ER+ breast cancer cells were determined using a total cholesterol content assay kit (Solarbio, Beijing, China) according to the manufacturer’s instructions. Briefly, cells (5 × 10^6^) suspended in 1 mL extraction buffer were sonicated (300 W, 2 s/3 s, 3 min) on ice and centrifuged at 10,000 × *g* for 10 min at 4 °C. Thereafterm the supernatant was collected for further detection using BioTek Synergy HTX microplate reader at 500 nm.

### Immunoprecipitation (IP) and mass spectrometry (IP-MS)

HEK293T cells stably expressing FLAG-KDM5B were generated by transfecting cells with FLAG-tagged KDM5B plasmid for 48 h. Cell lysate was applied to anti-FLAG immunoaffinity columns to elute the FLAG protein complex according to the manufacturer’s instructions. Fractions of the bed volume were collected, resolved using SDS-PAGE, and silver-stained. Thereafter, proteins were excised from the gel and subjected to liquid chromatography tandem mass spectrometry (LC-MS/MS) and data analysis. Detailed mass spectrometry data are presented in Table S4.

### GST pull-down assay

GST-fused proteins were expressed in BL21 *Escherichia coli* and purified with 30 µL glutathione sepharose 4B beads. In vitro transcription and translation experiments were performed with rabbit reticulocyte lysate (TNT systems; Promega) according to the manufacturer’s instructions. GST-fusion proteins were mixed with in vitro transcribed/translated products and incubated at 4 °C for 1 h. After five washes with binding buffer, the proteins were resuspended in the loading buffer and subjected to SDS-PAGE. Protein interactions were detected via immunoblotting.

### RNA sequencing (RNA-seq)

Total RNA was extracted from MCF-7 cells transfected with shSCR or shKDM5B using TRIzol reagent. Thereafter, the samples were sequenced using the Illumina NovaSeq 6000 platform by Lc-Bio Technology (Hangzhou, Zhejiang, China). Raw reads were mapped to the reference human genome (*Homo_sapiens*. GRCh38.100.chr) using HISAT2 software. Gene expression levels were represented as fragments per kilobase per million mapped fragments (FPKM) and analyzed using featureCounts. DEGs were identified using DESeq2 (*p* < 0.05, |Fold Change| > 1.5). Kyoto Encyclopedia of Genes and Genomes (KEGG) pathway analysis and gene set enrichment analysis (GSEA) were performed using the R package clusterProfiler. Raw sequencing data are deposited at https://www.ncbi.nlm.nih.gov/geo/query/ acc.cgi?acc=GSE286884.

### Chromatin immunoprecipitation (ChIP), ChIP-qPCR, and Re-ChIP

For ChIP experiments, MCF-7 cells (1 × 10^7^) were collected and cross-linked with 1% formaldehyde, sonicated, pre-cleared, and incubated with 2 µg ChIP-grade antibody for each reaction. Thereafter, Dynabeads™ Protein G (15 μl) was added to the mixture for another 4 h and incubated at 4 °C under gentle shaking. The beads were washed sequentially with specific low- and high-salt buffers and the enriched DNA was purified with the NucleoSpin Gel and PCR Clean-up Kit. The target sequence expression was detected using PerfectStart® Green qPCR SuperMix. The primer sequences used for ChIP-qPCR are listed in Table S5. For Re-ChIP assays, bead eluates from the first immunoprecipitation were incubated with 20 mM dithiothreitol (DTT) at 37 °C for 30 min and diluted at a ratio of 1:50 in ChIP dilution buffer followed by re-immunoprecipitation with secondary antibodies. The final elution step was performed using 1% SDS in Tris–EDTA buffer (pH 8.0).

### ChIP sequencing

Approximately 5 × 10^7^ MCF-7 cells were used for each ChIP sequencing. Chromatin DNA was precipitated using either normal goat IgG (control) or polyclonal antibodies against KDM5B or CUL4B. DNA was purified using Qiagen PCR purification kit (Qiagen, Hilden Germany) and in-depth whole-genome DNA sequencing was performed by LC-BIO Technology (Hangzhou, China) using Illumina NovaSeq 6000 platform. Raw sequencing data were aligned to the human reference genome (GRch38) using Bowtie2 and peaks were called using MACS2 software. Enriched binding peaks were generated after filtering using the control IgG. Additionally, the genomic distribution of KDM5B and CUL4B binding sites was analyzed using ChIPseeker, an R package for ChIP peak annotation, comparison, and visualization. KEGG pathway analysis was performed using the R package clusterProfiler. Notably, the ChIP-seq data are available at https://www.ncbi.nlm.nih.gov/geo/query/ acc.cgi?acc=GSE286885.

### In vivo tumorigenicity

All animal treatments and procedures were approved by the Animal Care Committee of Cancer Hospital and Institute, Chinese Academy of Medical Sciences (permit number: #NCC2022A056). MCF-7 cells (dilution: 2 × 10^6^, 1 × 10^6^,1 × 10^5^) infected with the vector or the KDM5B expression construct were inoculated into the left abdominal mammary fat pad of 6-week-old female NOD-SCID mice (*n* = 4 per group) to evaluate tumor growth. Similarly, T-47D cells (dilution: 5000, 1000, 500) stably transfected with shSCR or KDM5B knockdown lentiviruses were injected into mice. After 8 weeks, the mice were sacrificed following ethical guidelines. Tumor size was measured weekly using vernier caliper and tumor volume was calculated using the formula π/6 × length × width^2^. Additionally, the frequency of tumor formation was determined using extreme limiting dilution analysis (ELDA, https://bioinf.wehi.edu.au/software/elda/).

### Immunohistochemistry

Immunohistochemical assay was performed by Outdo Biotech (Shanghai, China) to detect KDM5B expression in breast cancer tissues. Briefly, tissue sections were maintained at 63 °C for 1 h and dewaxed with xylene and ethanol. Antigen retrieval was performed using DAKO Autostainer Link 48 (Agilent, Santa Clara, CA, USA). After three washes with PBST, the sections were incubated with specific primary antibodies overnight at 4 °C and subsequently incubated with secondary antibodies. Finally, the sections were developed using a DAB kit.

### Bioinformatics and statistical analysis

KDM5B expression levels were analyzed using public datasets downloaded from The Cancer Genome Atlas (TCGA) and the Gene Expression Omnibus (GEO) databases (GSE numbers are shown in the text). All bioinformatical and statistical analysis were performed using R Statistical Software (v4.2.0; R Core Team 2022) with in-house code. All experimental results represent at least three independent experiments. Significant differences were determined using a two-tailed unpaired Student’s t-test (two groups) or one-way analysis of variance (ANOVA) (multiple comparisons). Error bars indicate mean ± standard deviation (SD) or standard error of mean (SEM). Statistical significance was set at *p* < 0.05 (*p < 0.05, **p < 0.01, ***p < 0.001). ns indicates non-significance. Pearson’s correlation analysis was performed to determine the relationships between specific variables. Log-rank test was used for the statistical analysis of Kaplan–Meier survival plot.

## Supplementary information

SUPPLEMENTAL MATERIAL

Uncropped images

## Data Availability

All data needed to evaluate the conclusions in the paper are present in the paper and/or the Supplementary Materials. The original images are provided in the uncropped images of the Supplementary Materials. Additional data related to this paper may be requested from the authors.

## References

[1] Zhang Y, Yang H, Guo X, Rong N, Song Y, Xu Y, et al. The PHD1 finger of KDM5B recognizes unmodified H3K4 during the demethylation of histone H3K4me2/3 by KDM5B. Protein Cell. 2014;5:837–50. 24952722 10.1007/s13238-014-0078-4 PMC4225485

[2] Kooistra SM, Helin K. Molecular mechanisms and potential functions of histone demethylases. Nat Rev Mol Cell Biol. 2012;13:297–311. 22473470 10.1038/nrm3327

[3] Ohguchi Y, Ohguchi H. Diverse functions of KDM5 in cancer: transcriptional repressor or activator? Cancers. 2022;14:3270. 35805040 10.3390/cancers14133270 PMC9265395

[4] Howe FS, Fischl H, Murray SC, Mellor J. Is H3K4me3 instructive for transcription activation? Bioessays. 2017;39:1–12. 28004446 10.1002/bies.201600095

[5] Wang S, Meyer DH, Schumacher B. H3K4me2 regulates the recovery of protein biosynthesis and homeostasis following DNA damage. Nat Struct Mol Biol. 2020;27:1165–77. 33046905 10.1038/s41594-020-00513-1

[6] Tumber A, Nuzzi A, Hookway ES, Hatch SB, Velupillai S, Johansson C, et al. Potent and selective KDM5 inhibitor stops cellular demethylation of H3K4me3 at transcription start sites and proliferation of MM1S myeloma cells. Cell Chem Biol. 2017;24:371–80. 28262558 10.1016/j.chembiol.2017.02.006 PMC5361737

[7] Gong F, Miller KM. Histone methylation and the DNA damage response. Mutat Res Rev Mutat Res. 2019;780:37–47. 31395347 10.1016/j.mrrev.2017.09.003 PMC6690396

[8] Collins BE, Sweatt JD, Greer CB. Broad domains of histone 3 lysine 4 trimethylation are associated with transcriptional activation in CA1 neurons of the hippocampus during memory formation. Neurobiol Learn Mem. 2019;161:149–57. 31002880 10.1016/j.nlm.2019.04.009 PMC6541021

[9] Wang, H, & Helin, K Roles of H3K4 methylation in biology and disease. Trends Cell Biol*.* 2024;35:115–28. 10.1016/j.tcb.2024.06.001 38909006

[10] He R, Xhabija B, Gopi LK, Kurup JT, Xu Z, Liu Z, et al. H3K4 demethylase KDM5B regulates cancer cell identity and epigenetic plasticity. Oncogene. 2022;41:2958–72. 35440714 10.1038/s41388-022-02311-z PMC9426628

[11] Yoo J, Kim GW, Jeon YH, Kim JY, Lee SW, Kwon SH. Drawing a line between histone demethylase KDM5A and KDM5B: their roles in development and tumorigenesis. Exp Mol Med. 2022;54:2107–17. 36509829 10.1038/s12276-022-00902-0 PMC9794821

[12] Li CY, Wang W, Leung CH, Yang GJ, Chen J. KDM5 family as therapeutic targets in breast cancer: Pathogenesis and therapeutic opportunities and challenges. Mol Cancer. 2024;23:109. 38769556 10.1186/s12943-024-02011-0 PMC11103982

[13] Yamamoto S, Wu Z, Russnes HG, Takagi S, Peluffo G, Vaske C, et al. JARID1B is a luminal lineage-driving oncogene in breast cancer. Cancer Cell. 2014;25:762–77. 24937458 10.1016/j.ccr.2014.04.024 PMC4079039

[14] Fu YD, Huang MJ, Guo JW, You YZ, Liu HM, Huang LH, et al. Targeting histone demethylase KDM5B for cancer treatment. Eur J Med Chem. 2020;208:112760. 32883639 10.1016/j.ejmech.2020.112760

[15] Zheng YC, Chang J, Wang LC, Ren HM, Pang JR, Liu HM. Lysine demethylase 5B (KDM5B): a potential anti-cancer drug target. Eur J Med Chem. 2019;161:131–40. 30343192 10.1016/j.ejmech.2018.10.040

[16] Jose A, Shenoy GG, Sunil Rodrigues G, Kumar NAN, Munisamy M, Thomas L et al. Histone demethylase KDM5B as a therapeutic target for cancer therapy. Cancers*.* 2020;12:2121. 10.3390/cancers12082121 PMC7465382 32751840

[17] DiCiaccio B, Seehawer M, Li Z, Patmanidis A, Bui T, Foidart P, et al. ZBTB7A is a modulator of KDM5-driven transcriptional networks in basal breast cancer. Cell Rep. 2024;43:114991. 39570746 10.1016/j.celrep.2024.114991 PMC11694571

[18] Klein BJ, Piao L, Xi Y, Rincon-Arano H, Rothbart SB, Peng D, et al. The histone-H3K4-specific demethylase KDM5B binds to its substrate and product through distinct PHD fingers. Cell Rep. 2014;6:325–35. 24412361 10.1016/j.celrep.2013.12.021 PMC3918441

[19] Qin L, Song Y, Zhang F, Wang R, Zhou L, Jin S, et al. CRL4B complex-mediated H2AK119 monoubiquitination restrains Th1 and Th2 cell differentiation. Cell Death Differ. 2023;30:1488–502. 37024604 10.1038/s41418-023-01155-8 PMC10244459

[20] Ji Q, Hu H, Yang F, Yuan J, Yang Y, Jiang L, et al. CRL4B interacts with and coordinates the SIN3A-HDAC complex to repress CDKN1A and drive cell cycle progression. J Cell Sci. 2014;127:4679–91. 25189618 10.1242/jcs.154245

[21] Leng S, Huang W, Chen Y, Yang Y, Feng D, Liu W, et al. SIRT1 coordinates with the CRL4B complex to regulate pancreatic cancer stem cells to promote tumorigenesis. Cell Death Differ. 2021;28:3329–43. 34163012 10.1038/s41418-021-00821-z PMC8630059

[22] Hu H, Yang Y, Ji Q, Zhao W, Jiang B, Liu R, et al. CRL4B catalyzes H2AK119 monoubiquitination and coordinates with PRC2 to promote tumorigenesis. Cancer Cell. 2012;22:781–95. 23238014 10.1016/j.ccr.2012.10.024

[23] Yin X, Teng X, Ma T, Yang T, Zhang J, Huo M, et al. RUNX2 recruits the NuRD(MTA1)/CRL4B complex to promote breast cancer progression and bone metastasis. Cell Death Differ. 2022;29:2203–17. 35534547 10.1038/s41418-022-01010-2 PMC9613664

[24] Yang T, Huang W, Ma T, Yin X, Zhang J, Huo M, et al. The PRMT6/PARP1/CRL4B complex regulates the circadian clock and promotes breast tumorigenesis. Adv Sci. 2023;10:e2202737. 10.1002/advs.202202737 PMC10190619 36941223

[25] Hanahan D. Hallmarks of cancer: new dimensions. Cancer Discov. 2022;12:31–46. 35022204 10.1158/2159-8290.CD-21-1059

[26] Luo J, Yang H, Song BL. Mechanisms and regulation of cholesterol homeostasis. Nat Rev Mol Cell Biol. 2020;21:225–45. 31848472 10.1038/s41580-019-0190-7

[27] Liu W, Chakraborty B, Safi R, Kazmin D, Chang CY, McDonnell DP. Dysregulated cholesterol homeostasis results in resistance to ferroptosis increasing tumorigenicity and metastasis in cancer. Nat Commun. 2021;12:5103. 34429409 10.1038/s41467-021-25354-4 PMC8385107

[28] Wu Q, Ishikawa T, Sirianni R, Tang H, McDonald JG, Yuhanna IS, et al. 27-hydroxycholesterol promotes cell-autonomous, ER-positive breast cancer growth. Cell Rep. 2013;5:637–45. 24210818 10.1016/j.celrep.2013.10.006 PMC3950897

[29] Nelson ER, Wardell SE, Jasper JS, Park S, Suchindran S, Howe MK, et al. 27-hydroxycholesterol links hypercholesterolemia and breast cancer pathophysiology. Science. 2013;342:1094–8. 24288332 10.1126/science.1241908 PMC3899689

[30] Goicoechea L, Conde de la Rosa L, Torres S, García-Ruiz C, Fernández-Checa JC. Mitochondrial cholesterol: metabolism and impact on redox biology and disease. Redox Biol. 2023;61:102643. 36857930 10.1016/j.redox.2023.102643 PMC9989693

[31] Meaney S. Epigenetic regulation of cholesterol homeostasis. Front Genet. 2014;5:311. 25309573 10.3389/fgene.2014.00311 PMC4174035

[32] Catchpole S, Spencer-Dene B, Hall D, Santangelo S, Rosewell I, Guenatri M, et al. PLU-1/JARID1B/KDM5B is required for embryonic survival and contributes to cell proliferation in the mammary gland and in ER+ breast cancer cells. Int J Oncol. 2011;38:1267–77. 21369698 10.3892/ijo.2011.956

[33] Bakir B, Chiarella AM, Pitarresi JR, Rustgi AK. EMT, MET, plasticity, and tumor metastasis. Trends Cell Biol. 2020;30:764–76. 32800658 10.1016/j.tcb.2020.07.003 PMC7647095

[34] Stewart MH, Albert M, Sroczynska P, Cruickshank VA, Guo Y, Rossi DJ, et al. The histone demethylase Jarid1b is required for hematopoietic stem cell self-renewal in mice. Blood. 2015;125:2075–8. 25655602 10.1182/blood-2014-08-596734 PMC4467872

[35] Cellot S, Hope KJ, Chagraoui J, Sauvageau M, Deneault É, MacRae T, et al. RNAi screen identifies Jarid1b as a major regulator of mouse HSC activity. Blood. 2013;122:1545–55. 23777767 10.1182/blood-2013-04-496281 PMC5289888

[36] Wang Y, Zhang H, Chen Y, Sun Y, Yang F, Yu W, et al. LSD1 is a subunit of the NuRD complex and targets the metastasis programs in breast cancer. Cell. 2009;138:660–72. 19703393 10.1016/j.cell.2009.05.050

[37] Ren Z, Kim A, Huang YT, Pi WC, Gong W, Yu X, et al. A PRC2-Kdm5b axis sustains tumorigenicity of acute myeloid leukemia. Proc Natl Acad Sci USA. 2022;119:e2122940119. 35217626 10.1073/pnas.2122940119 PMC8892512

[38] Cavadini S, Fischer ES, Bunker RD, Potenza A, Lingaraju GM, Goldie KN, et al. Cullin-RING ubiquitin E3 ligase regulation by the COP9 signalosome. Nature. 2016;531:598–603. 27029275 10.1038/nature17416

[39] Luo M, Bao L, Chen Y, Xue Y, Wang Y, Zhang B, et al. ZMYND8 is a master regulator of 27-hydroxycholesterol that promotes tumorigenicity of breast cancer stem cells. Sci Adv. 2022;8:eabn5295. 35857506 10.1126/sciadv.abn5295 PMC9286501

[40] Hinohara K, Wu HJ, Vigneau S, McDonald TO, Igarashi KJ, Yamamoto KN, et al. KDM5 histone demethylase activity links cellular transcriptomic heterogeneity to therapeutic resistance. Cancer Cell. 2018;34:939–953.e939. 30472020 10.1016/j.ccell.2018.10.014 PMC6310147

[41] Lin CS, Lin YC, Adebayo BO, Wu A, Chen JH, Peng YJ, et al. Silencing JARID1B suppresses oncogenicity, stemness and increases radiation sensitivity in human oral carcinoma. Cancer Lett. 2015;368:36–45. 26184998 10.1016/j.canlet.2015.07.003

[42] Facompre ND, Harmeyer KM, Sole X, Kabraji S, Belden Z, Sahu V, et al. JARID1B enables transit between distinct states of the stem-like cell population in oral cancers. Cancer Res. 2016;76:5538–49. 27488530 10.1158/0008-5472.CAN-15-3377 PMC5026599

[43] Xiang Y, Zhu Z, Han G, Ye X, Xu B, Peng Z, et al. JARID1B is a histone H3 lysine 4 demethylase up-regulated in prostate cancer. Proc Natl Acad Sci USA. 2007;104:19226–31. 18048344 10.1073/pnas.0700735104 PMC2148272

[44] Wang D, Han S, Peng R, Jiao C, Wang X, Yang X, et al. Depletion of histone demethylase KDM5B inhibits cell proliferation of hepatocellular carcinoma by regulation of cell cycle checkpoint proteins p15 and p27. J Exp Clin Cancer Res. 2016;35:37. 26911146 10.1186/s13046-016-0311-5 PMC4766611

[45] Shen H, Xu W, Guo R, Rong B, Gu L, Wang Z, et al. Suppression of enhancer overactivation by a RACK7-histone demethylase complex. Cell. 2016;165:331–42. 27058665 10.1016/j.cell.2016.02.064 PMC4826479

[46] Shen HF, Zhang WJ, Huang Y, He YH, Hu GS, Wang L, et al. The dual function of KDM5C in both gene transcriptional activation and repression promotes breast cancer cell growth and tumorigenesis. Adv Sci (Weinh). 2021;8:2004635. 33977073 10.1002/advs.202004635 PMC8097366

[47] Zhang SM, Cai WL, Liu X, Thakral D, Luo J, Chan LH, et al. KDM5B promotes immune evasion by recruiting SETDB1 to silence retroelements. Nature. 2021;598:682–7. 34671158 10.1038/s41586-021-03994-2 PMC8555464

[48] Wu L, Cao J, Cai WL, Lang SM, Horton JR, Jansen DJ, et al. KDM5 histone demethylases repress immune response via suppression of STING. PLoS Biol. 2018;16:e2006134. 30080846 10.1371/journal.pbio.2006134 PMC6095604

[49] Li G, Kanagasabai T, Lu W, Zou MR, Zhang SM, Celada SI, et al. KDM5B is essential for the hyperactivation of PI3K/AKT signaling in prostate tumorigenesis. Cancer Res. 2020;80:4633–43. 32868382 10.1158/0008-5472.CAN-20-0505 PMC8034842

[50] Horton JR, Engstrom A, Zoeller EL, Liu X, Shanks JR, Zhang X, et al. Characterization of a linked jumonji domain of the KDM5/JARID1 family of histone H3 lysine 4 demethylases. J Biol Chem. 2016;291:2631–46. 26645689 10.1074/jbc.M115.698449 PMC4742734

[51] Huang B, Song BL, Xu C. Cholesterol metabolism in cancer: mechanisms and therapeutic opportunities. Nat Metab. 2020;2:132–41. 32694690 10.1038/s42255-020-0174-0

[52] Voisin M, de Medina P, Mallinger A, Dalenc F, Huc-Claustre E, Leignadier J, et al. Identification of a tumor-promoter cholesterol metabolite in human breast cancers acting through the glucocorticoid receptor. Proc Natl Acad Sci USA. 2017;114:E9346–e9355. 29078321 10.1073/pnas.1707965114 PMC5676900

[53] Ning S, Liu C, Wang K, Cai Y, Ning Z, Li M, et al. NUCB2/Nesfatin-1 drives breast cancer metastasis through the up-regulation of cholesterol synthesis via the mTORC1 pathway. J Transl Med. 2023;21:362. 37277807 10.1186/s12967-023-04236-x PMC10243030

[54] Umetani M, Domoto H, Gormley AK, Yuhanna IS, Cummins CL, Javitt NB, et al. 27-hydroxycholesterol is an endogenous SERM that inhibits the cardiovascular effects of estrogen. Nat Med. 2007;13:1185–92. 17873880 10.1038/nm1641

[55] DuSell CD, Umetani M, Shaul PW, Mangelsdorf DJ, McDonnell DP. 27-hydroxycholesterol is an endogenous selective estrogen receptor modulator. Mol Endocrinol. 2008;22:65–77. 17872378 10.1210/me.2007-0383 PMC2194632

[56] Nelson ER. The significance of cholesterol and its metabolite, 27-hydroxycholesterol in breast cancer. Mol Cell Endocrinol. 2018;466:73–80. 28919300 10.1016/j.mce.2017.09.021 PMC5854519

[57] El Roz A, Bard JM, Huvelin JM, Nazih H. LXR agonists and ABCG1-dependent cholesterol efflux in MCF-7 breast cancer cells: relation to proliferation and apoptosis. Anticancer Res. 2012;32:3007–13. 22753765

[58] Lam M, Mast N, Pikuleva IA. Drugs and scaffold that inhibit cytochrome P450 27A1 in vitro and in vivo. Mol Pharmacol. 2018;93:101–8. 29192124 10.1124/mol.117.110742 PMC5749491

[59] Baek AE, Yu YA, He S, Wardell SE, Chang CY, Kwon S, et al. The cholesterol metabolite 27 hydroxycholesterol facilitates breast cancer metastasis through its actions on immune cells. Nat Commun. 2017;8:864. 29021522 10.1038/s41467-017-00910-z PMC5636879

