# Supplementary material for: KDM5B cooperates with CRL4B complex to promote the tumorigenesis of ER+ breast cancer via regulating cholesterol metabolism
Source: Cell Death Dis. 2026 Feb 7;17(1):207. doi: 10.1038/s41419-026-08438-1 (PMC12894857; doi:10.1038/s41419-026-08438-1)
Supplement: Supplementary file 1 — SUPPLEMENTAL MATERIAL [file 41419_2026_8438_MOESM1_ESM.docx]

**Supplementary Information for**

**KDM5B cooperates with CRL4B complex to promote the tumorigenesis of ER+ breast cancer via regulating cholesterol metabolism**

Yunkai Yang^1,4^, Tianyang Gao^1, 4^, Baowen Yuan^1, 4^, Xinhui Hao^1^, Miaomiao Huo^1^, Ting Hu^1^, Tianyu Ma^1^, Min Zhang^1^, Die Zhang^1^, Xu Teng^2^, Hefen Yu^2^, Wei Huang^2,*^, Jingyao Zhang^1,*^, Yan Wang^1,2,3,*^

^1^ State Key Laboratory of Molecular Oncology, National Cancer Center/National Clinical Research Center for Cancer/Cancer Hospital, Chinese Academy of Medical Sciences and Peking Union Medical College, Beijing 100021, China

^2^ Department of Biochemistry and Molecular Biology, School of Basic Medical Sciences, Capital Medical University, Beijing 100069, China

^3^ Institute of Cancer Research, Henan Academy of Innovations in Medical Sciences, Zhengzhou, Henan, 450000, China

^4^These authors contributed equally: Yunkai Yang, Tianyang Gao, Baowen Yuan.

* Corresponding author:

Wei Huang

Email: weihuang@ccmu.edu.cn

Jingyao Zhang

zhangjingyao@pumc.edu.cn

Yan Wang

Email: yanwang@cicams.ac.cn

**Supplementary Figures**


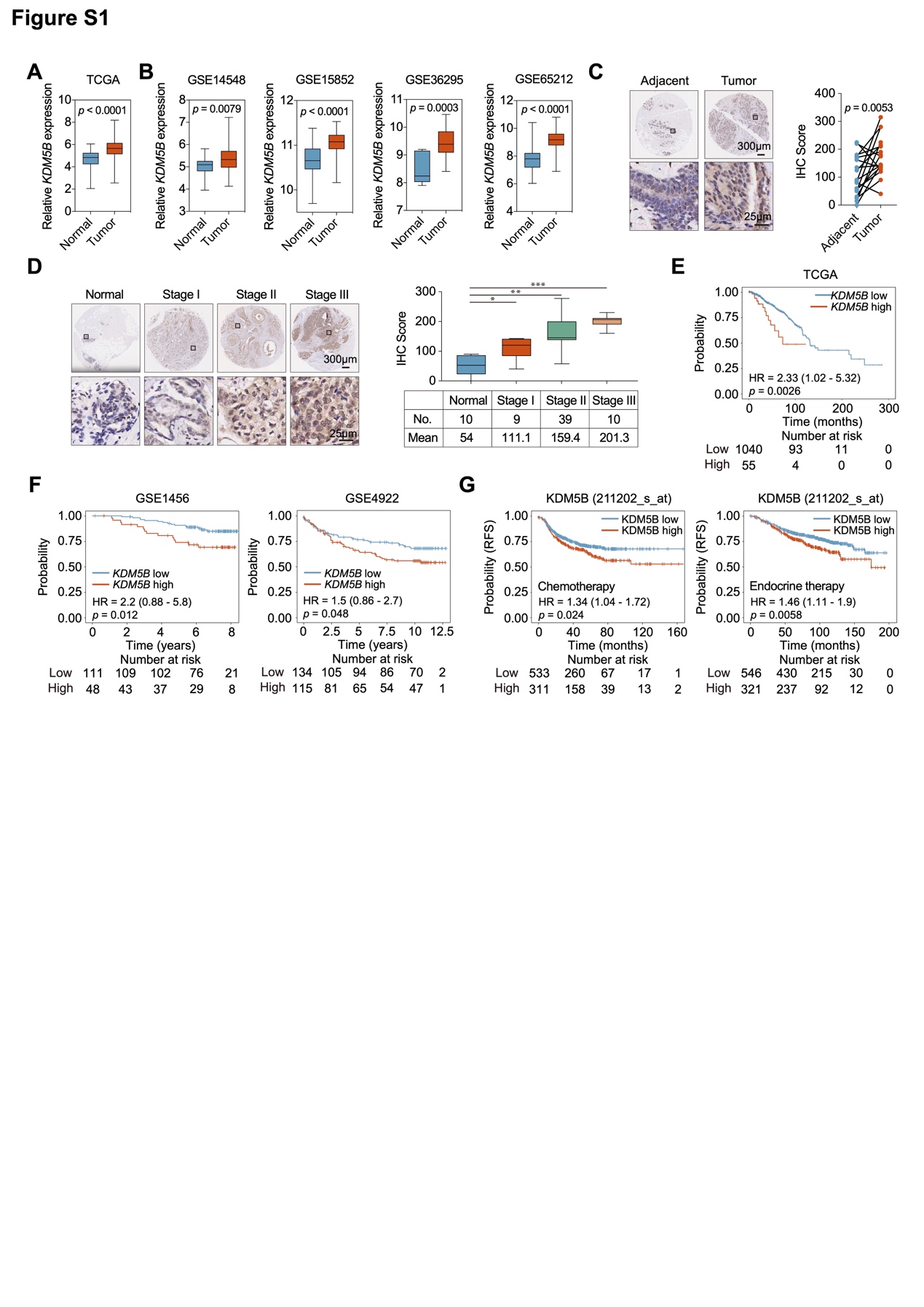


Fig. S1. KDM5B was upregulated and predicted poor prognosis in breast cancer. (A–B) KDM5B expression in breast cancer and normal tissues based on TCGA and GEO analysis. (C) 18 paired breast cancer specimen and adjacent normal tissues were collected for immunohistochemistry (IHC) to detect KDM5B expression. (D) IHC was performed to examine KDM5B levels in different stages of breast. (E–F) The TCGA and GEO datasets were used to analyze the correlation between KDM5B expression and survival rate of patients with breast cancer. (G) Correlation between KDM5B expression and the survival rate of patients with breast cancer receiving chemotherapy or endocrine therapy was analyzed using Kaplan-Meier plotter (https://kmplot.com/analysis/). Data were shown as mean ± SD. Data were analyzed using two-tailed unpaired t-test or one-way ANOVA. **p* < 0.05, ***p* < 0.01, ****p* < 0.001.


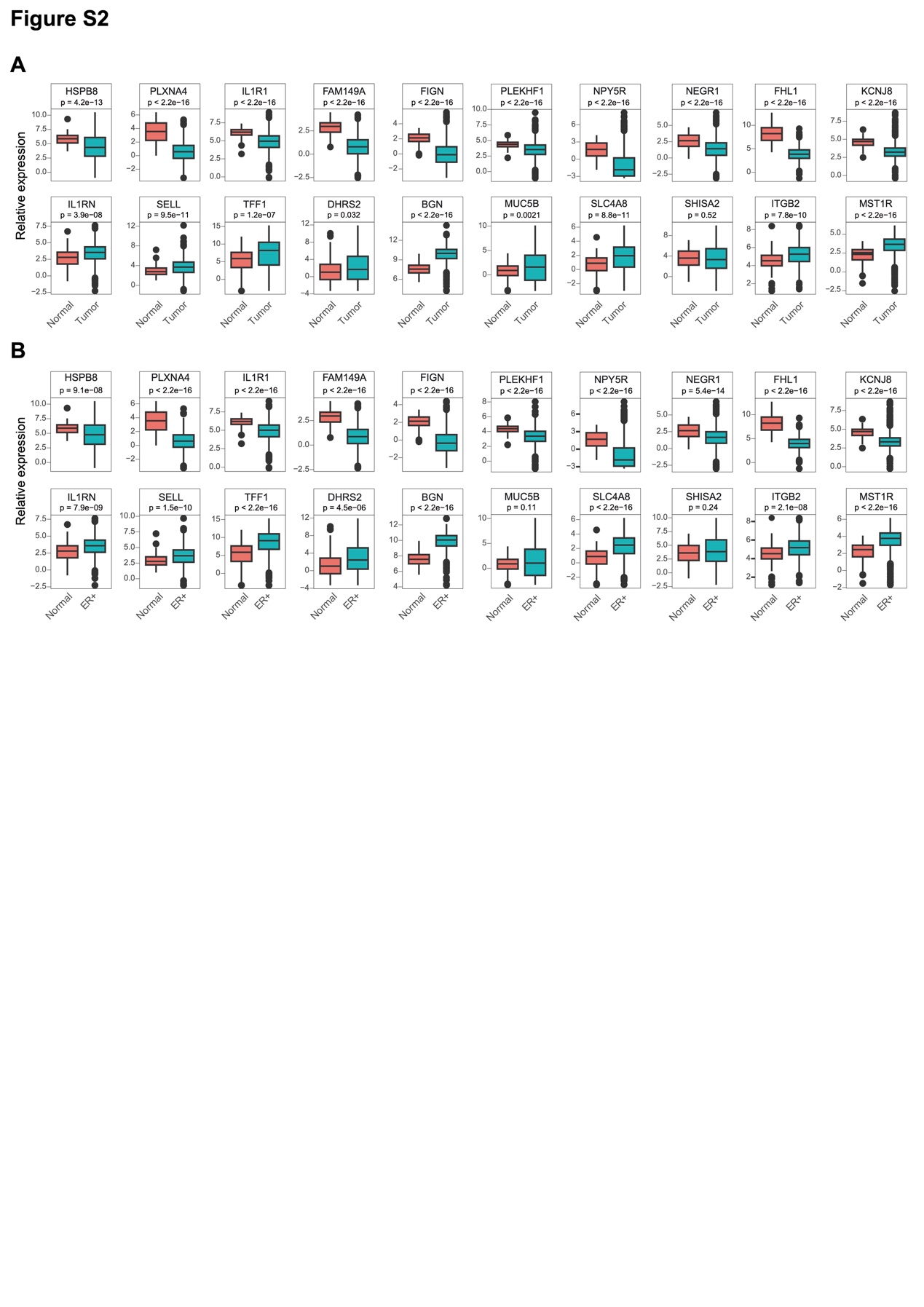


Fig. S2. The expression of potential TSGs and oncogenes in breast cancer. (A–B) The levels of potential TSGs and oncogenes, identified by RNA-seq data, were evaluated in breast cancer (A) and ER+ breast cancer (B) using TCGA dataset.


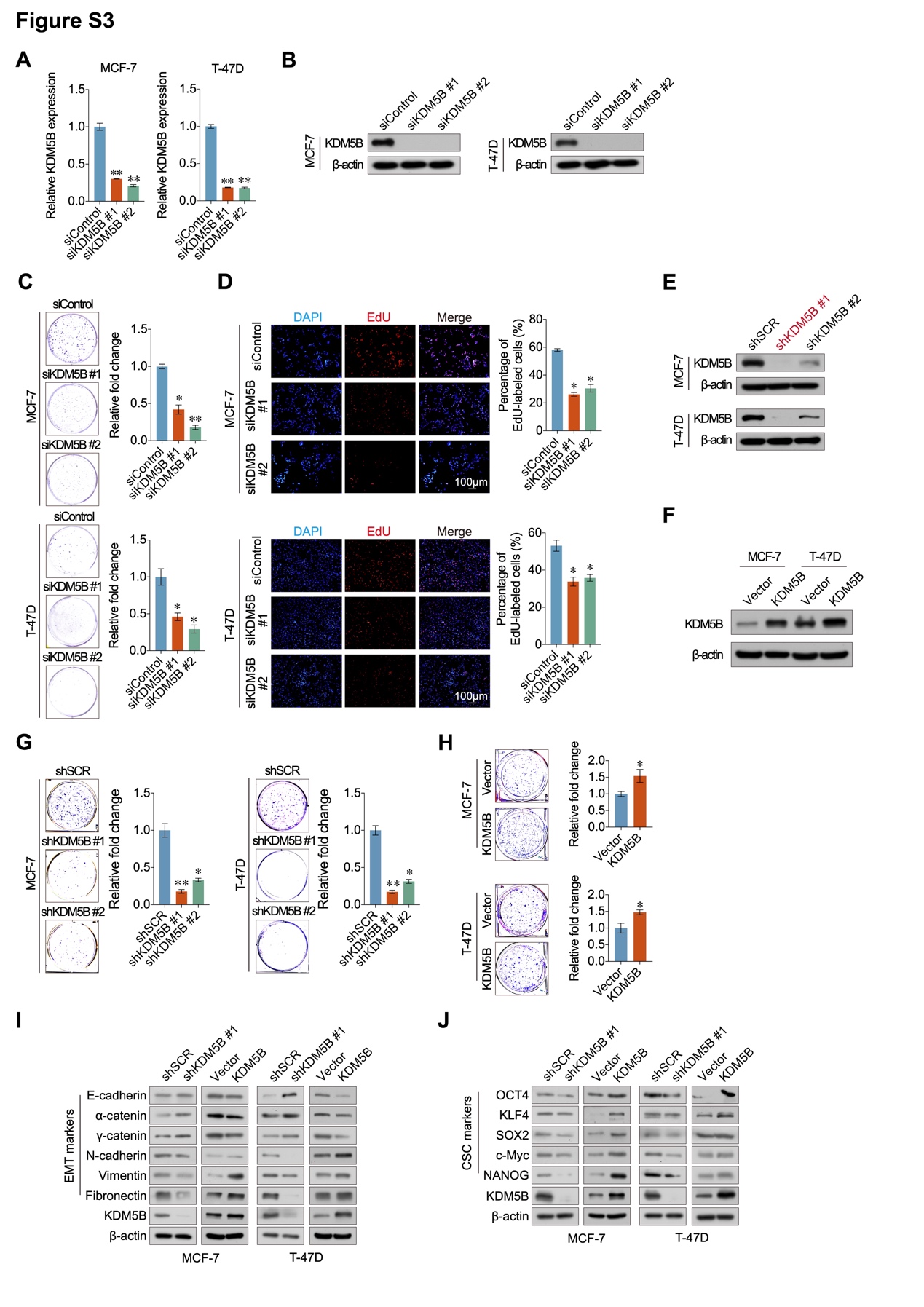


Fig. S3. KDM5B promoted cell proliferation and expression of epithelial-mesenchymal cell transition (EMT) and stemness markers in ER+ breast cancer cells. (A–B) mRNA (A) and protein (B) levels of KDM5B in MCF-7 and T-47D cells transfected with control siRNA or siRNAs targeting KDM5B. (C–D) The proliferation ability MCF-7 and T-47D cells transfected with control siRNA or siRNAs targeting KDM5B was evaluated using colony formation assay (C) and EdU experiment (D). (E–F) KDM5B protein levels were detected in MCF-7 and T-47D cells using western blotting following KDM5B knockdown and overexpression. (G–H) Colony formation assays were used to examine the proliferation of ER+ breast cancer cells following KDM5B knockdown and overexpression. (I–J) The expression of EMT and CSC markers were examined in MCF-7 and T-47D cells following KDM5B knockdown and overexpression. Data were shown as mean ± SD. Data were analyzed using two-tailed unpaired t-test or one-way ANOVA. **p* < 0.05, ***p* < 0.01.


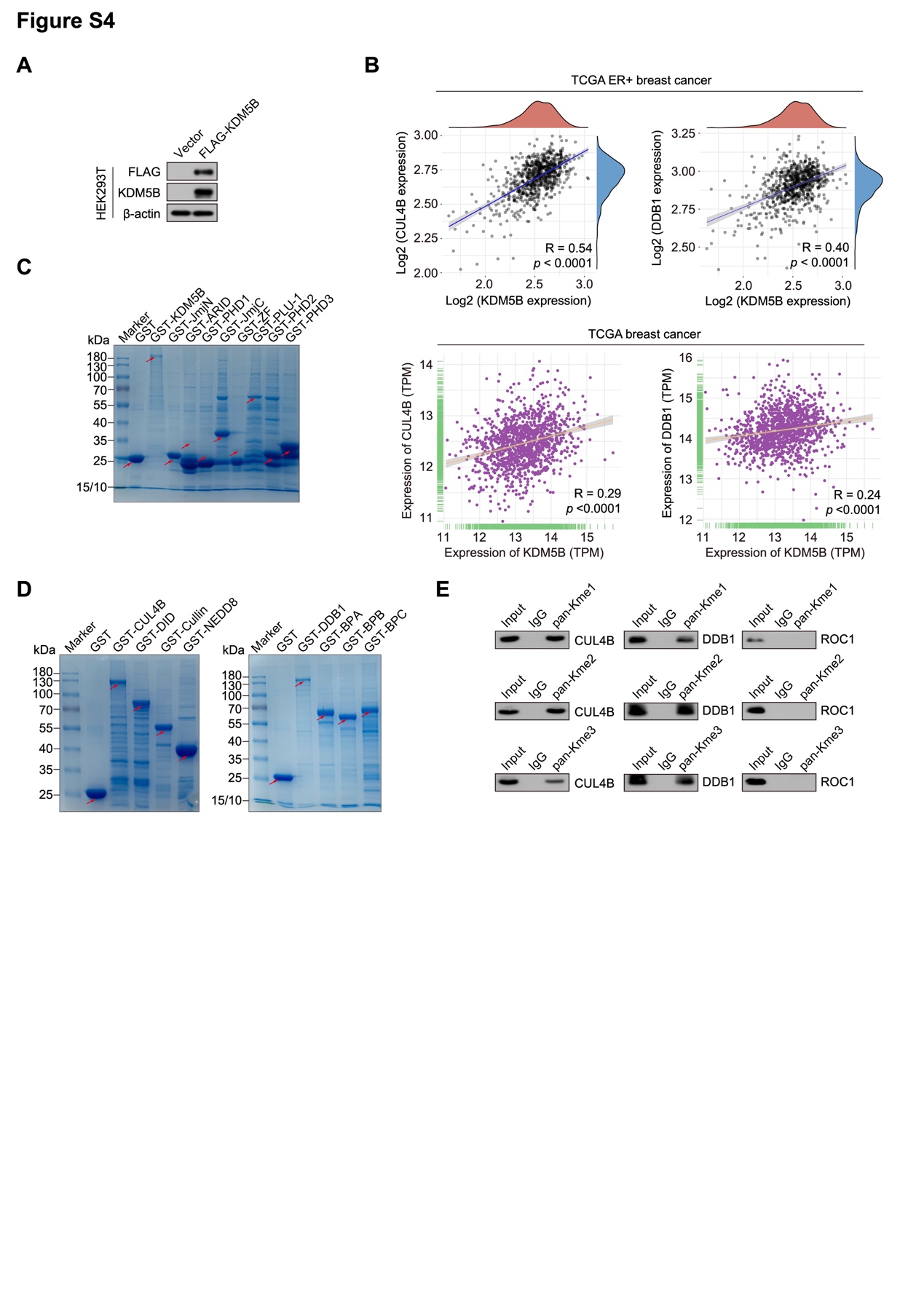


Fig. S4. (A) The FLAG-tagged KDM5B was overexpressed and detected in HEK293T cells using western blotting. (B) The correlation of KDM5B with CUL4B or DDB1 was analyzed in breast cancer using TCGA dataset. (C–D) The GST-fused whole proteins and fragmented proteins were verified using Coomassie brilliant blue staining. (E) The lysine methylation status of CUL4B, DDB1 and ROC1.


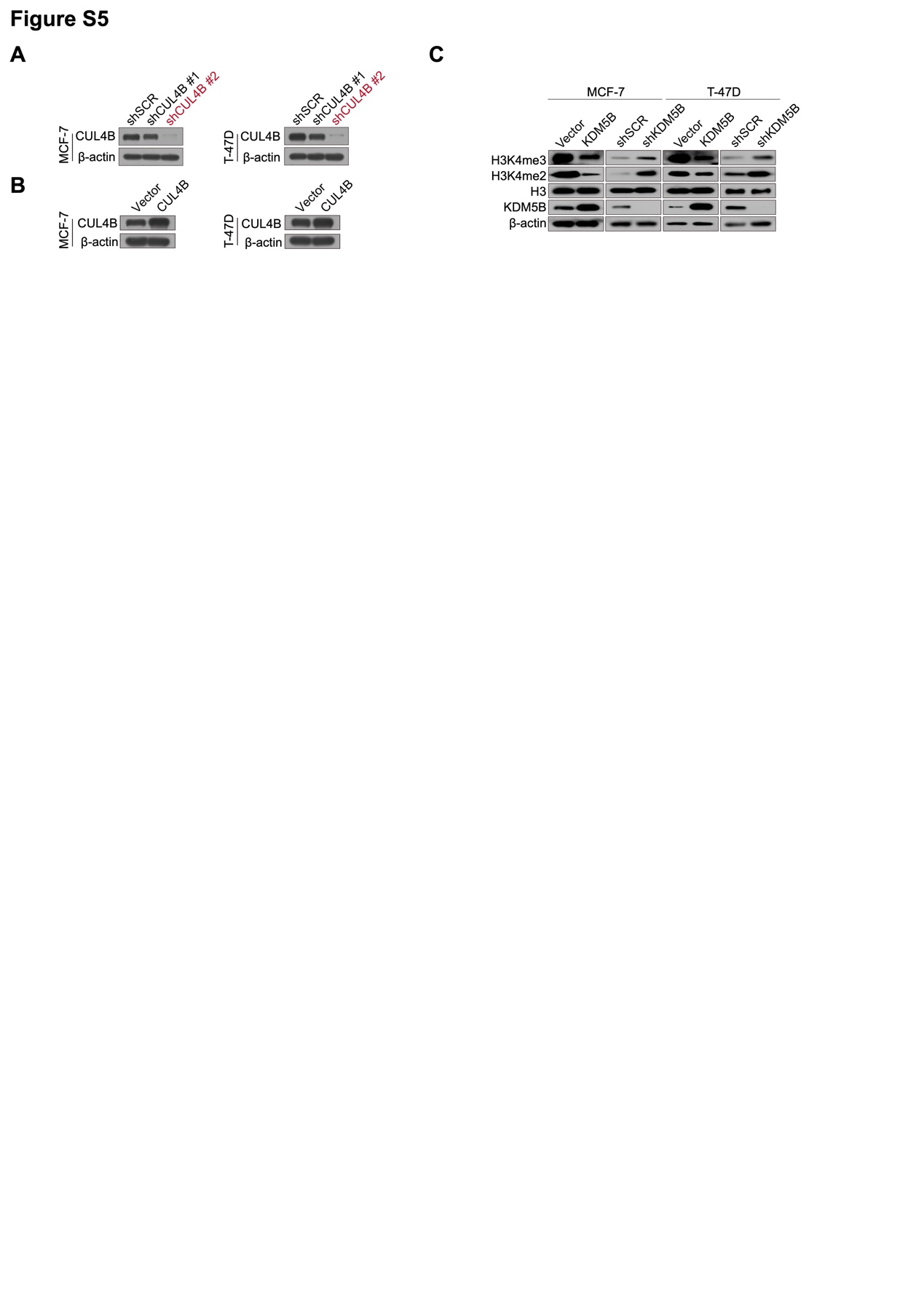


Fig. S5. Construction of CUL4B overexpression and knockdown breast cancer cells. (A–B) CUL4B expression in MCF-7 and T-47D cells with or without CUL4B-knockdown (A) or -overexpression (B). (C) Verifying the demethylation activity of KDM5B on H3K4me2/3.


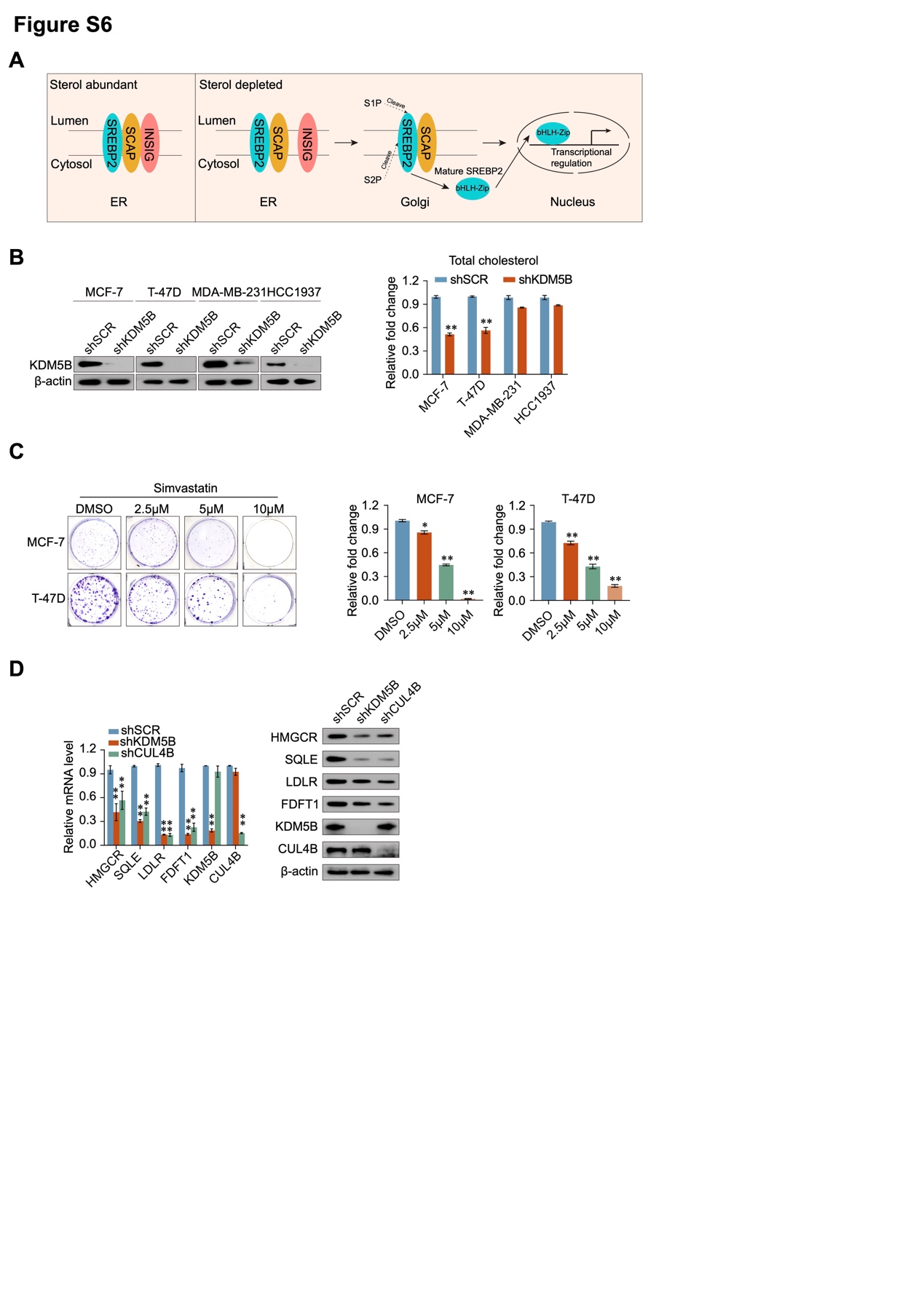


Fig. S6. (A) Schematic of cholesterol biosynthesis. (B) Total cholesterol levels in KDM5B deficient breast cancer cells were examined. (C) The proliferation status of breast cancer cells under simvastatin treatment. (D) The expression levels of genes related to the cholesterol synthesis pathway. Data were shown as mean ± SD. The data were analyzed by two-tailed unpaired t-test or one-way ANOVA. **p* < 0.05, ***p* < 0.01.

**Supplementary Tables**

**Table S1. The sequences of siRNAs and shRNAs.**

| **siRNA or shRNA** | **Sequences (5'–3')** |
| --- | --- |
| siControl | UUCUCCGAACGUGUCACGUTT |
| siKDM5B #1 | CCGUAAAUUGGGAGUGAUUTT |
| siKDM5B #2 | CAUCAAGCAAGAACCUAUUTT |
| siINSIG1 | GCGUCACGGUGGGGAACAUTT |
| siINSIG2 | GAUAGAUCUAGAAGUGGUUTT |
| shSCR | TTCTCCGAACGTGTCACGT |
| shKDM5B #1 | CGAGATGGAATTAACAGTCTT |
| shKDM5B #2 | CCCACCAATTTGGAAGGCATT |
| shCUL4B #1 | GCCACGTACCGATACAGAAGA |
| shCUL4B #2 | GGATTCATTGGATAGCGTTCT |
| shDDB1 | TCCACTAGATCGCGATAATAA |

**Table S2. The plasmids used in this study.**

| **Plasmids** | **Sources** | **Vectors** |
| --- | --- | --- |
| FLAG-KDM5B | This study | p3×FLAG-CMV |
| GST-KDM5B | This study | pGEX-4T-3 |
| GST-CUL4B | This study | pGEX-4T-3 |
| GST-DDB1 | This study | pGEX-4T-3 |
| GST-ROC1 | This study | pGEX-4T-3 |
| GST-JmjN | This study | pGEX-4T-3 |
| GST-ARID | This study | pGEX-4T-3 |
| GST-PHD1 | This study | pGEX-4T-3 |
| GST-JmjC | This study | pGEX-4T-3 |
| GST-ZF | This study | pGEX-4T-3 |
| GST-PLU-1 | This study | pGEX-4T-3 |
| GST-PHD2 | This study | pGEX-4T-3 |
| GST-PHD3 | This study | pGEX-4T-3 |
| GST-DID | This study | pGEX-4T-3 |
| GST-Cullin | This study | pGEX-4T-3 |
| GST-NEDD8 | This study | pGEX-4T-3 |
| GST-BPA | This study | pGEX-4T-3 |
| GST-BPB | This study | pGEX-4T-3 |
| GST-BPC | This study | pGEX-4T-3 |

**Table S3. The primer sequences used in RT-qPCR.**

| **Gene** | **Forward primer (5'–3')** | **Reverse primer (5'–3')** |
| --- | --- | --- |
| *KDM5B* | CCATAGCCGAGCAGACTGG | GGATACGTGGCGTAAAATGAAGT |
| *CUL4B* | ACTCCTCCTTTACAACCCAGG | TCTTCGCATCAAACCCTACAAAC |
| *DDB1* | ACCGGACACTTTACTTCGGC | TCGGCGGTGACCACATAGA |
| *IL1RN* | CATTGAGCCTCATGCTCTGTT | CGCTGTCTGAGCGGATGAA |
| *SELL* | ACCCAGAGGGACTTATGGAAC | GCAGAATCTTCTAGCCCTTTGC |
| *TFF1* | CCCCGTGAAAGACAGAATTGT | GGTGTCGTCGAAACAGCAG |
| *DHRS2* | ATGGGAATGAGTCTCTCTGGAA | CGTTGACGTAGCTGGCATC |
| *BGN* | CAGTGGCTTTGAACCTGGAG | GGGAGGTCTTTGGGGATGC |
| *MUC5B* | GCCCACATCTCCACCTATGAT | GCAGTTCTCGTTGTCCGTCA |
| *SLC4A8* | GACGGCGTCCTCAGCTATC | GCATCCGAACTCCCACATACA |
| *SHISA2* | TCAAGGGCAGTATCTGCATCC | CCGTCCATGAAAGGTGGCA |
| *ITGB2* | AAGTGACGCTTTACCTGCGAC | AAGCATGGAGTAGGAGAGGTC |
| *MST1R* | CTTTGACGTGAAGTACGTGGT | CGTATGGCTACAAACACAGCAC |
| *HSPB8* | CTCCTGCCACTACCCAAGC | GGCCAAGAGGCTGTCAAGT |
| *PLXNA4* | GTCATTTGTCACATTCCGAGGA | GCTTGTAAATCCGATTGACGGC |
| *IL1R1* | GGCCAGTTGAGTGACATTGCT | TGTGATGAGGGTACTCCTTCTTT |
| *FAM149A* | ACCCGCCCCAGATTCATCA | CTGTCAGCAAAATAGGCAGGT |
| *FIGN* | GTAGCACCAGTGTTTATGGCT | GTAGGCTTCAACTTTGTGGGC |
| *PLEKHF1* | CCTCTGCTACCGCGAACTG | CTCGTCGGAGTCATCGTCATC |
| *NPY5R* | CGGTAAACTTCCTCATAGGCAAT | AACATCCACTGATCCAGCAAG |
| *NEGR1* | GCTTGTTGCTCGAACCAGTG | CCCCTTTTCTGACCATCATGTT |
| *FHL1* | TGCTGCCTGAAATGCTTTGAC | GCCAGAAGCGGTTCTTATAGTG |
| *KCNJ8* | CTCTTCGCTATCATGTGGTGG | GACCTGACATTAGTCACACACAC |
| *BMAL1* | AAGGGAAGCTCACAGTCAGAT | GGACATTGCGTTGCATGTTGG |
| *FOXO1* | TCGTCATAATCTGTCCCTACACA | CGGCTTCGGCTCTTAGCAAA |
| *GATA3* | GCCCCTCATTAAGCCCAAG | TTGTGGTGGTCTGACAGTTCG |
| *CDKN1A* | TGTCCGTCAGAACCCATGC | AAAGTCGAAGTTCCATCGCTC |
| *CDKN1B* | TAATTGGGGCTCCGGCTAACT | TGCAGGTCGCTTCCTTATTCC |
| *INSIG1* | CCTGGCATCATCGCCTGTT | AGAGTGACATTCCTCTGGATCTG |
| *INSIG2* | CTTGATGATTCGAGGAGTAGTGC | CAGGTGGAAAGAGCGTCACAT |
| *RHOB* | CTGCTGATCGTGTTCAGTAAGG | TCAATGTCGGCCACATAGTTC |
| *CYP27A1* | CGGCAACGGAGCTTAGAGG | GGCATAGCCTTGAACGAACAG |
| *HMGCR* | TGATTGACCTTTCCAGAGCAAG | CTAAAATTGCCATTCCACGAGC |
| *LDLR* | TCTGCAACATGGCTAGAGACT | TCCAAGCATTCGTTGGTCCC |
| *SQLE* | GGCATTGCCACTTTCACCTAT | GGCCTGAGAGAATATCCGAGAAG |
| *FDFT1* | CCACCCCGAAGAGTTCTACAA | TGCGACTGGTCTGATTGAGATA |
| *ACTB* | CATGTACGTTGCTATCCAGGC | CTCCTTAATGTCACGCACGAT |

**Table S4. Mass spectrometry analysis of KDM5B-containing protein complex.**

| **Description** | **Peptides (95%)** | **Score** | **%Coverage** | **%Coverage [50]** | **%Coverage [95]** |
| --- | --- | --- | --- | --- | --- |
| KDM5B | 197 | 51.77 | 41.76999927 | 37.18000054 | 33.23000073 |
| HSPA8 | 56 | 41.93 | 47.20999897 | 46.27999961 | 42.41000116 |
| HSPA1A | 36 | 27.69 | 44.76999938 | 44.76999938 | 38.38 |
| PRMT5 | 33 | 33.14 | 38.78000081 | 35.01000106 | 30.61000109 |
| HSPA5 | 21 | 27.84 | 36.23999953 | 26.60999894 | 25.08000135 |
| KRT10 | 19 | 29.36 | 30.30999899 | 30.30999899 | 26.71000063 |
| KIF11 | 16 | 29.8 | 15.99999964 | 15.44000059 | 14.76999968 |
| SLC25A5 | 16 | 17.49 | 43.61999929 | 42.62000024 | 36.91000044 |
| IQGAP1 | 11 | 19.92 | 12.48999983 | 10.62000021 | 6.880000234 |
| DDX21 | 10 | 19.84 | 21.07000053 | 18.26000065 | 14.04999942 |
| MCM3 | 9 | 16.53 | 9.776999801 | 9.776999801 | 9.776999801 |
| PPM1B | 9 | 14.6 | 25.88999867 | 24.6299997 | 19.61999983 |
| HNRNPU | 9 | 12.95 | 12.35999987 | 10.41999981 | 9.211999923 |
| HUWE1 | 7 | 14.38 | 2.19500009 | 2.19500009 | 1.669000089 |
| PSMD11 | 7 | 13.56 | 25.58999956 | 19.91000026 | 17.7699998 |
| VIM | 7 | 12.61 | 23.8199994 | 17.5999999 | 15.67000002 |
| ILF2 | 5 | 10.68 | 20.76999992 | 20.76999992 | 17.94999987 |
| PSMB5 | 5 | 10.32 | 28.90000045 | 26.6200006 | 23.56999964 |
| WRNIP1 | 5 | 7.96 | 11.87999994 | 9.172999859 | 8.120000362 |
| DDB1 | 4 | 8.36 | 6.491000205 | 4.825000092 | 3.421000019 |
| TRIM28 | 4 | 7.34 | 9.820000082 | 7.903999835 | 7.304999977 |
| RIOK1 | 3 | 4.74 | 6.689999998 | 6.689999998 | 5.105999857 |
| MTHFD1 | 2 | 2.88 | 5.561000109 | 4.278000072 | 2.246000059 |
| FLNA | 1 | 2.13 | 0.530500012 | 0.530500012 | 0.303100003 |
| RBBP4 | 1 | 2 | 3.058999963 | 3.058999963 | 3.058999963 |
| RBBP7 | 1 | 2 | 3.125 | 3.125 | 3.125 |
| CDK4 | 1 | 1.98 | 4.619999975 | 2.639999986 | 2.639999986 |
| PRMT1 | 1 | 1.78 | 5.816999823 | 3.047000058 | 3.047000058 |
| WDR26 | 1 | 1.59 | 4.030999914 | 2.945999987 | 1.394999959 |
| PARP1 | 1 | 1.42 | 1.676999964 | 0.986200012 | 0.986200012 |
| MATR3 | 1 | 0.81 | 3.542000055 | 2.006999962 | 0.944500044 |
| NLRP2 | 1 | 0.58 | 1.882999949 | 0.941599999 | 0.941599999 |
| MTA2 | 0 | 0.34 | 4.490999877 | 2.694999985 | 0 |

Footnotes: %Cov [95]: The number of amino acids identified with a confidence of more than 95% of peptide segments accounts for the proportion of the total number of protein amino acids. %Cov [50]: The number of amino acids identified with a confidence of more than 50% of peptide segments accounts for the proportion of the total number of protein amino acids.

**Table S5. The primer sequences used in ChIP-qPCR.**

| **Gene** | **Forward primer (5'–3')** | **Reverse primer (5'–3')** |
| --- | --- | --- |
| *BMAL1* | GCCGGGAAAGTGTTCGCT | GTCCTCTTCGGCCGAGTCAC |
| *FOXO1* | GGAGGACTTGACAGATCGCAG | TCTTTGCTGAACGACGTGGG |
| *GATA3* | CACTCCTACATGGACGCGG | GAGTTTCCGTAGTAGGGCGG |
| *CDKN1A* | TCACTACTCCCTCCAGCGG | CTGTGTGCTATTCCCGCCA |
| *CDKN1B* | TCGCCAGTCCATTTGATCAGC | AAAGACACAGACCCCGACGA |
| *INSIG1* | CCTGTTGGGTCTTTGGGACG | CAAGGTTAGACCCCCGTGC |
| *INSIG2* | GCCAGGGCTCAGCGTAGAAT | AAGTGCCACACAAGAAACCGT |
| *RHOB* | CGCATCCAAGCCTACGACTA | CCCTCATAGCACCTTGCAGC |
| *GAPDH* | GCACGTAGCTCAGGCCTCAAGAC | GACTGTCGAACAGGAGGAGCAGAG |
