# Supplementary material for: KDM5B cooperates with CRL4B complex to promote the tumorigenesis of ER+ breast cancer via regulating cholesterol metabolism
Source: Cell Death Dis. 2026 Feb 7;17(1):207. doi: 10.1038/s41419-026-08438-1 (PMC12894857; doi:10.1038/s41419-026-08438-1)
Supplement: Supplementary file 2 — Uncropped images [file 41419_2026_8438_MOESM2_ESM.docx]

**Uncropped original blots:**

**Figure 1B**


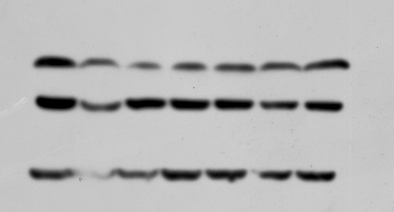


1B_β-actin


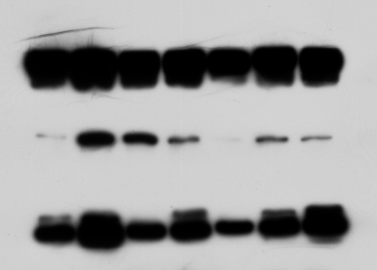


1B_KDM5B

**Figure 3**


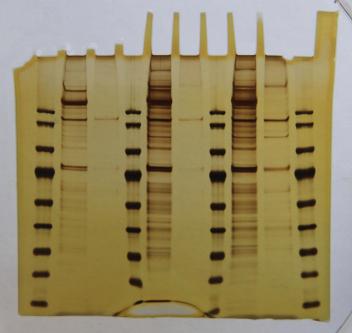


3A


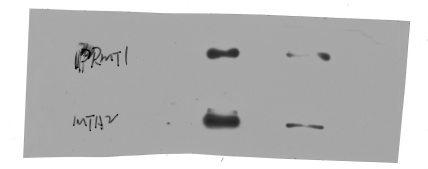


3B_PRMT1

3C_MTA2


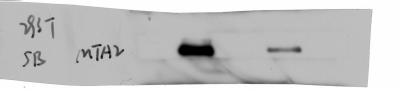


3B_MTA2


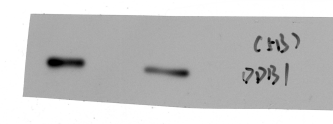


3B_DDB1


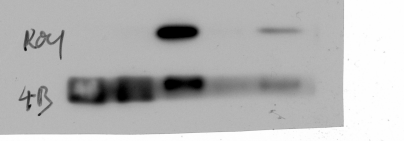


3B_ROC1

3B_CUL4B


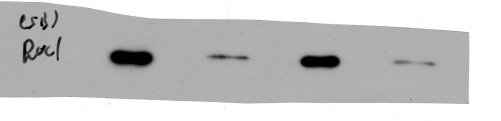


3C_ROC1


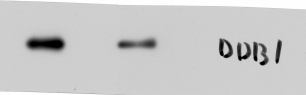


3C_DDB1


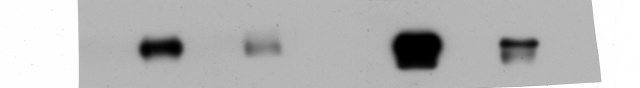


3C_CUL4B


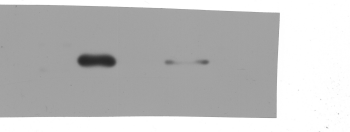


3C_PRMT1


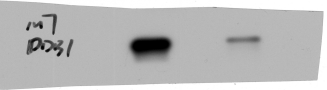


3E_MCF7_DDB1


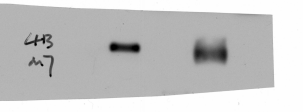


3E_MCF7_CUL4B


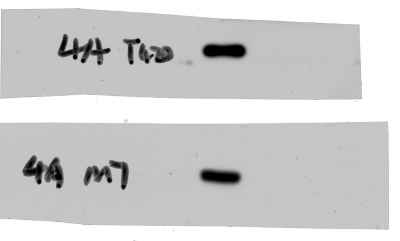


3E_T-47D_CUL4A


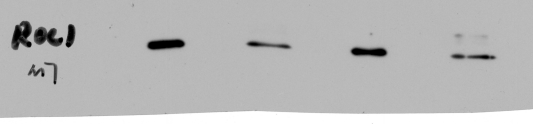


3E_MCF7_ROC1

3E_MCF7_CUL4A


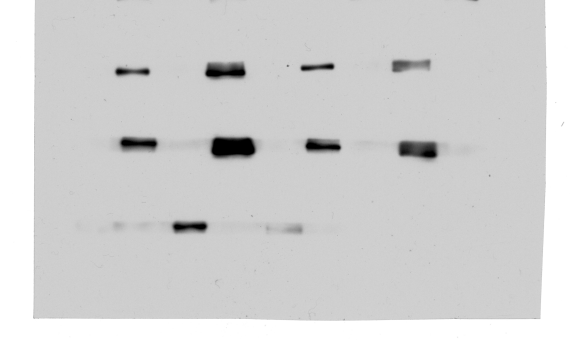


CUL4B

DDB1

ROC1

DDB1

3F_T-47D


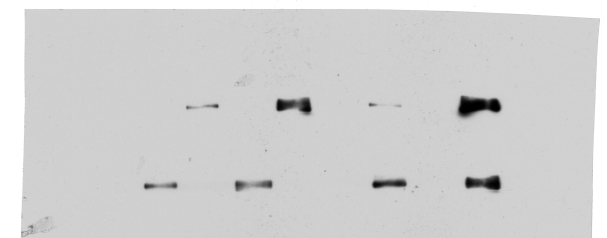


3F_T-47D_CUL4B

3F_MCF7_CUL4B

3F_MCF7_DDB1

3F_MCF7_ROC1

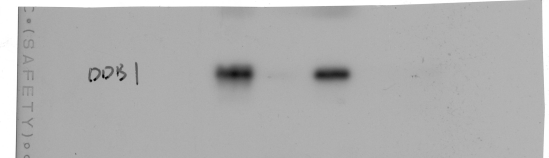


3H_right_panel_DDB1

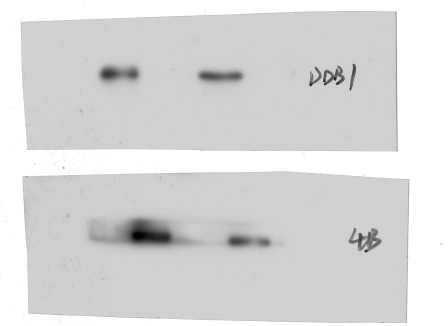


3H_left_panel_DDB1

3H_left_panel_CUL4B

3C_ROC1


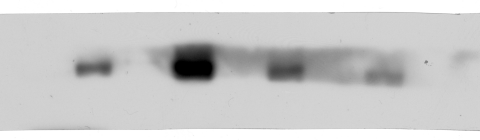


3H_right_panel_CUL4B

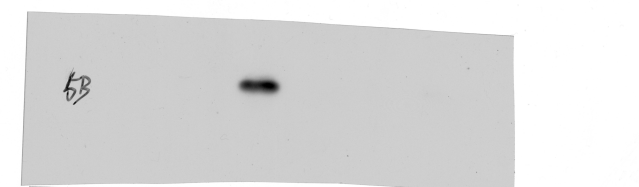


3H_right_panel_ROC1

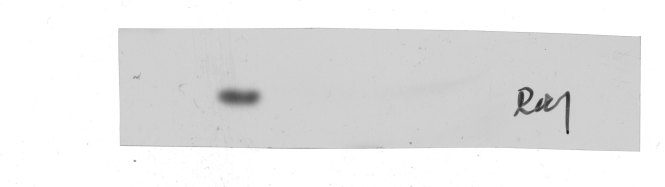


3H_left_panel_ROC1

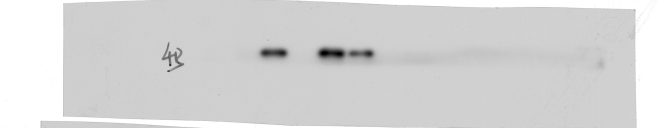


3I_CUL4B


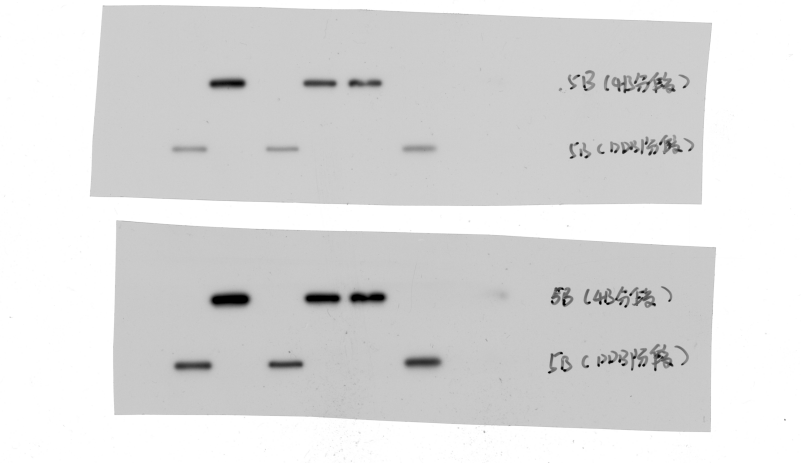


3J_up_KDM5B

3J_down_KDM5B


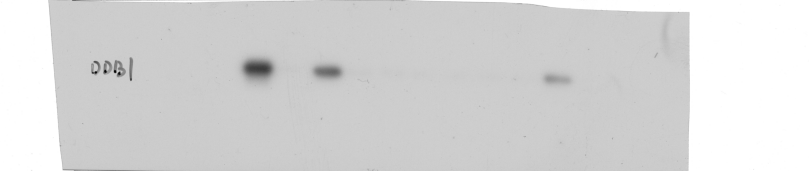


3I_DDB1


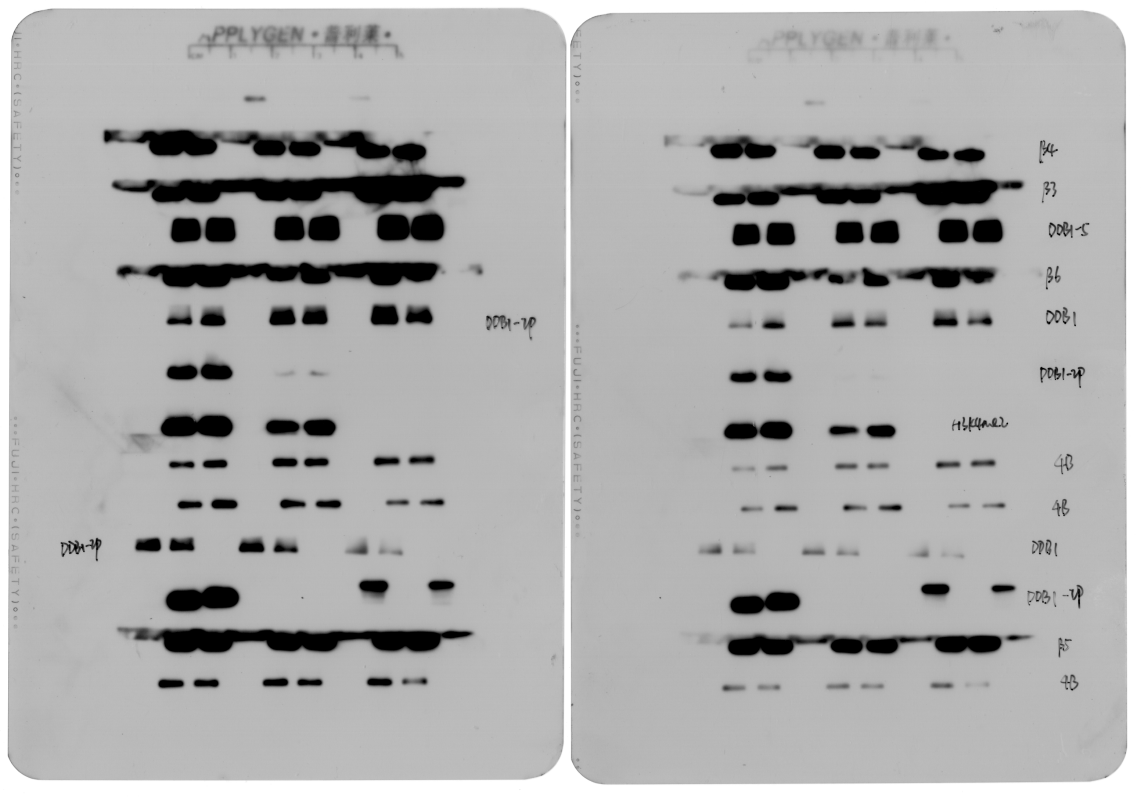


3K_left_CUL4B_1

3K_left_DDB1_1

3K_right_DDB1_2

3K_right_CUL4B_2

3K_right_DDB1_1

3K_right_CUL4B_1

3K_left_CUL4B_2


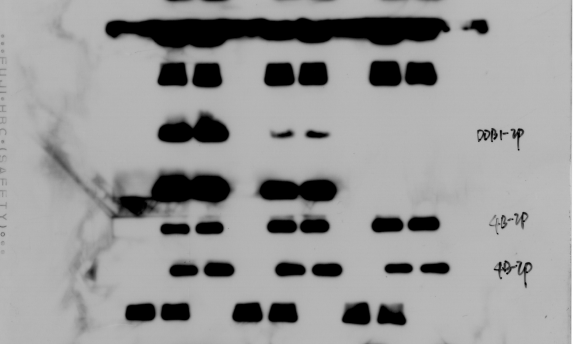

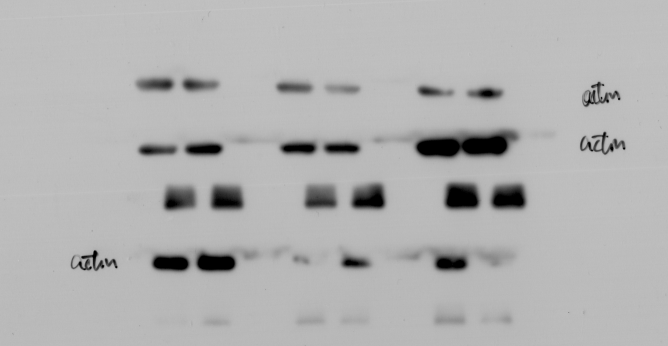


3K_right_actin

3K_left_actin

3K_left_DDB1_2


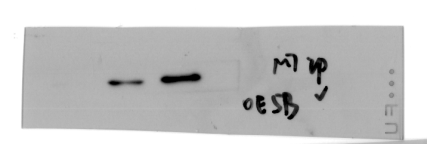

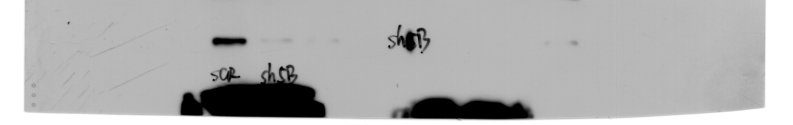


3K_right_KDM5B

3K_left_KDM5B


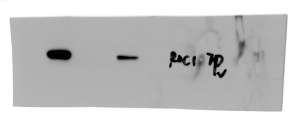


3F_HEK293T

ROC1

**Figure 4**


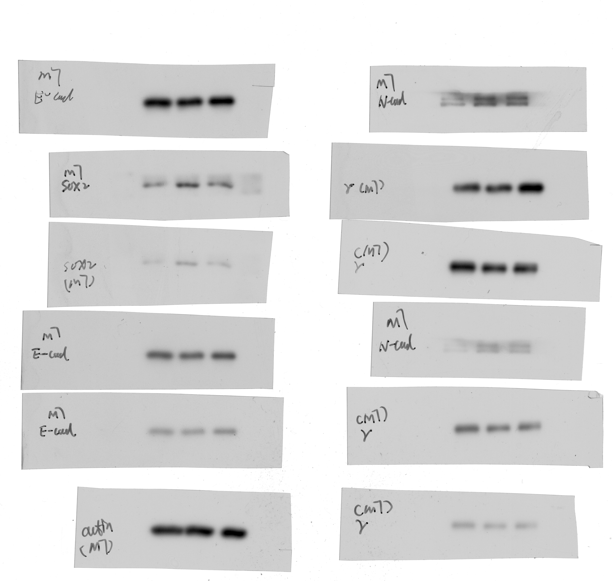

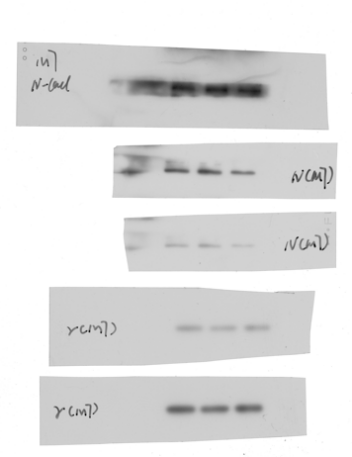

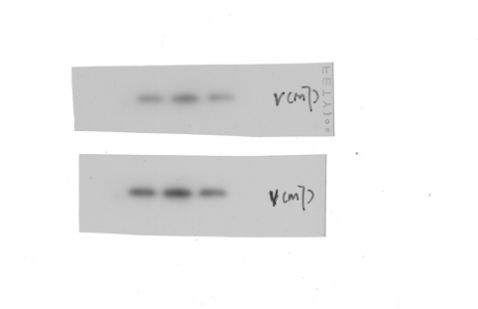

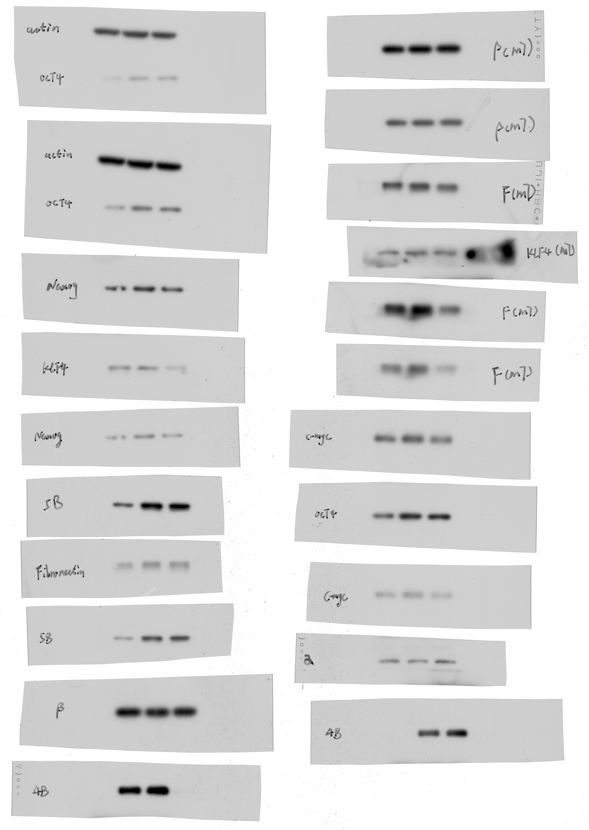


4G_left_MCF7_SOX2

4G_left_MCF7_E-cadherin

4G_left_MCF7_N-cadherin

4G_left_MCF7_γ-catenin

4G_left_MCF7_α-catenin

4G_left_MCF7_Vimentin

4G_left_MCF7_CUL4B

4G_left_MCF7_β-actin

4G_left_MCF7_KDM5B

4G_left_MCF7_OCT4

4G_left_MCF7_c-Myc

4G_left_MCF7_KLF4

4G_left_MCF7_NANOG

4G_left_MCF7_Fibronectin


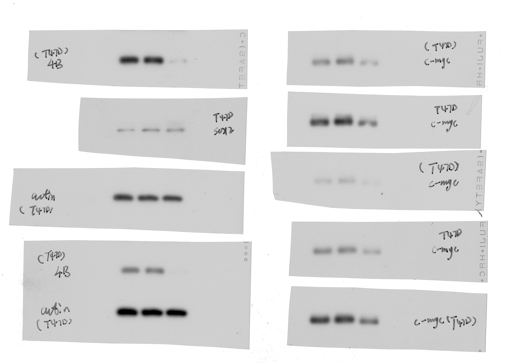

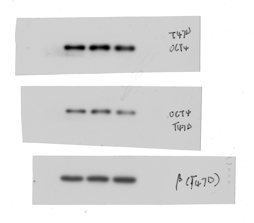

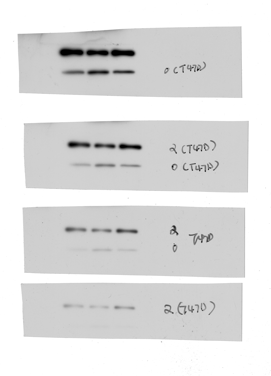

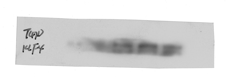

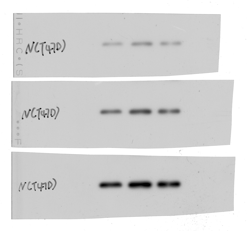

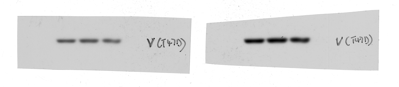

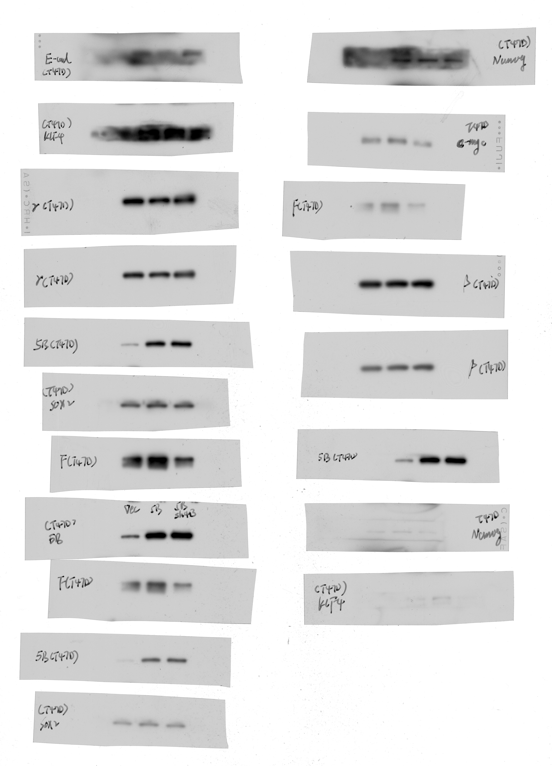


4G_left_T-47D_OCT4

4G_left_T-47D_KLF4

4G_left_T-47D_SOX2

4G_left_T-47D_c-Myc

4G_left_T-47D_NANOG

4G_left_T-47D_E-cadherin

4G_left_T-47D_α-catenin

4G_left_T-47D_γ-catenin

4G_left_T-47D_N-cadherin

4G_left_T-47D_Vimentin

4G_left_T-47D_Fibronectin

4G_left_T-47D_KDM5B

4G_left_T-47D_CUL4B

4G_left_T-47D_β-actin


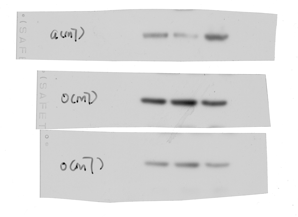

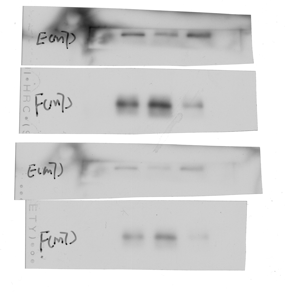

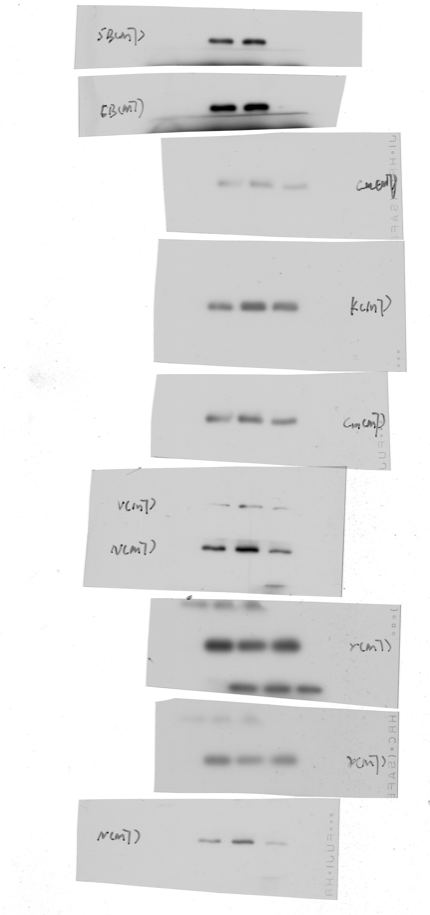

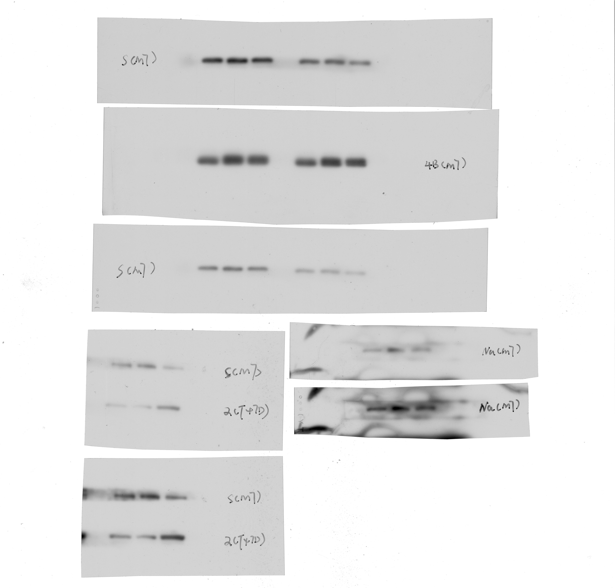


4G_right_MCF7_OCT4

4G_right_MCF7_KLF4

4G_right_MCF7_SOX2

4G_right_MCF7_c-Myc

4G_right_MCF7_NANOG

4G_right_MCF7_E-cadherin

4G_right_MCF7_α-catenin

4G_right_MCF7_γ-catenin

4G_right_MCF7_N-cadherin

4G_right_MCF7_Fibronectin

4G_right_MCF7_KDM5B

4G_right_MCF7_CUL4B

4G_right_MCF7_Vimentin


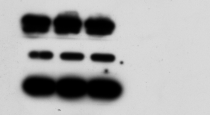


4G_right_MCF7_β-actin


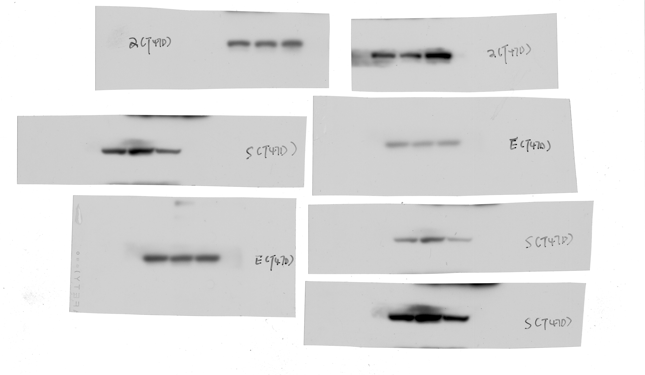

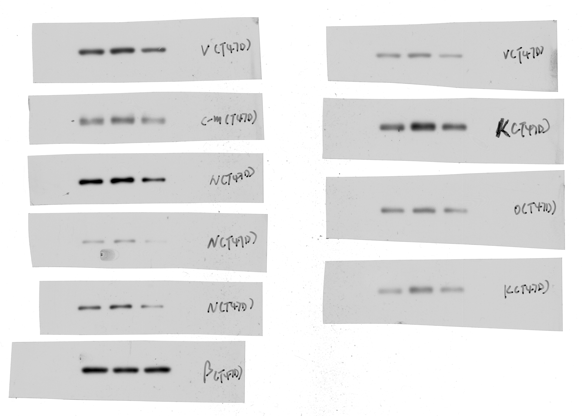

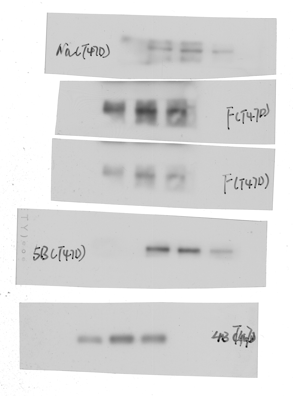

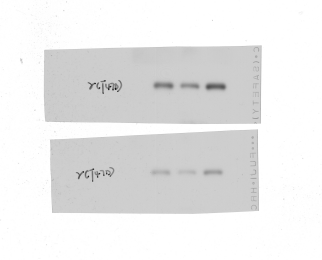


4G_right_T-47D_OCT4

4G_right_T-47D_KLF4

4G_right_T-47D_SOX2

4G_right_T-47D_c-Myc

4G_right_T-47D_NANOG

4G_right_T-47D_E-cadherin

4G_right_T-47D_α-catenin

4G_right_T-47D_γ-catenin

4G_right_T-47D_N-cadherin

4G_right_T-47D_Vimentin

4G_right_T-47D_Fibronectin

4G_right_T-47D_KDM5B

4G_right_T-47D_CUL4B

4G_right_T-47D_β-actin

**Figure 5**


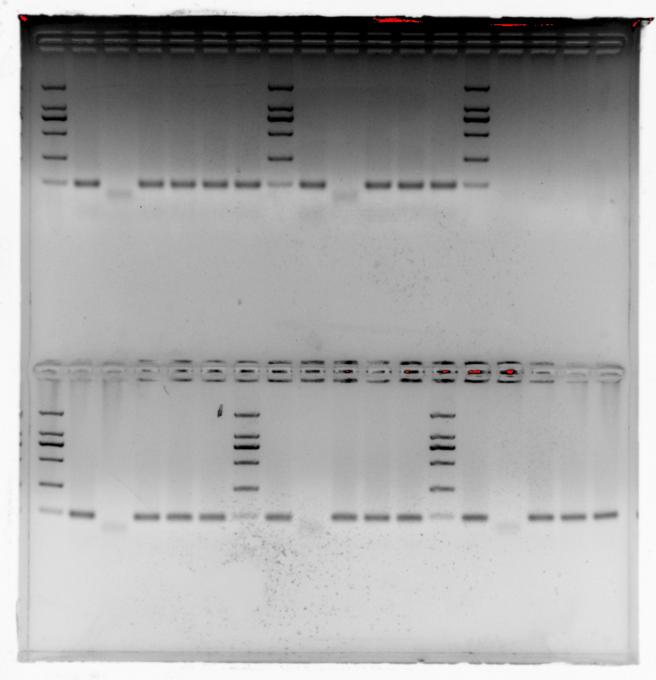


5G_INSIG1


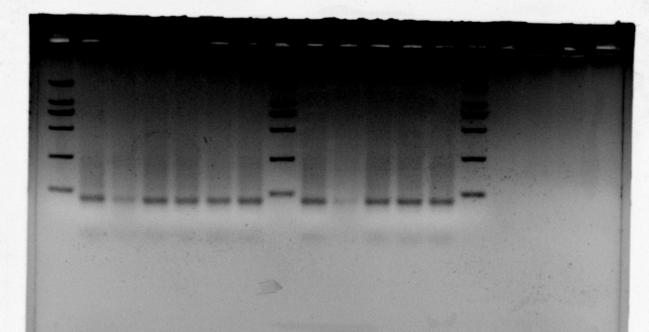


5G_INSIG2


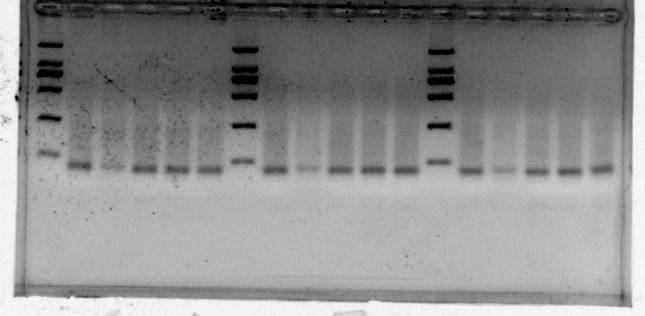


5G_INSIG2


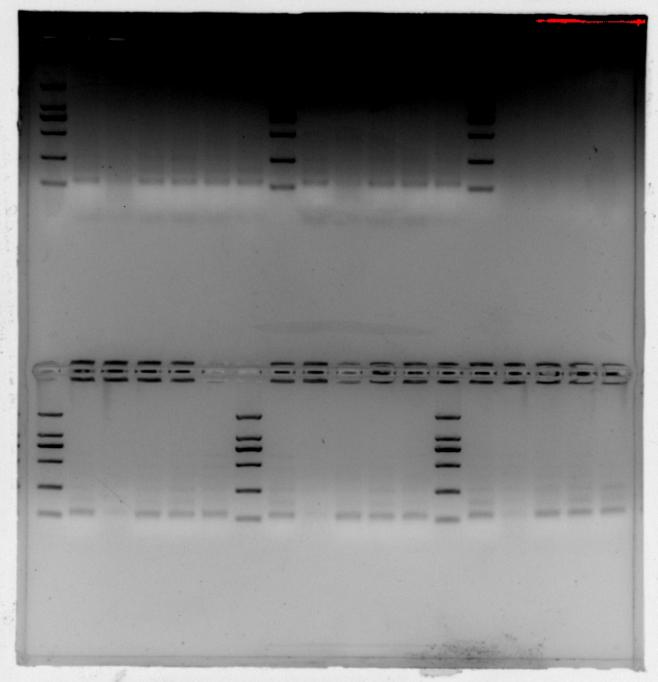


5G_CDKN1B


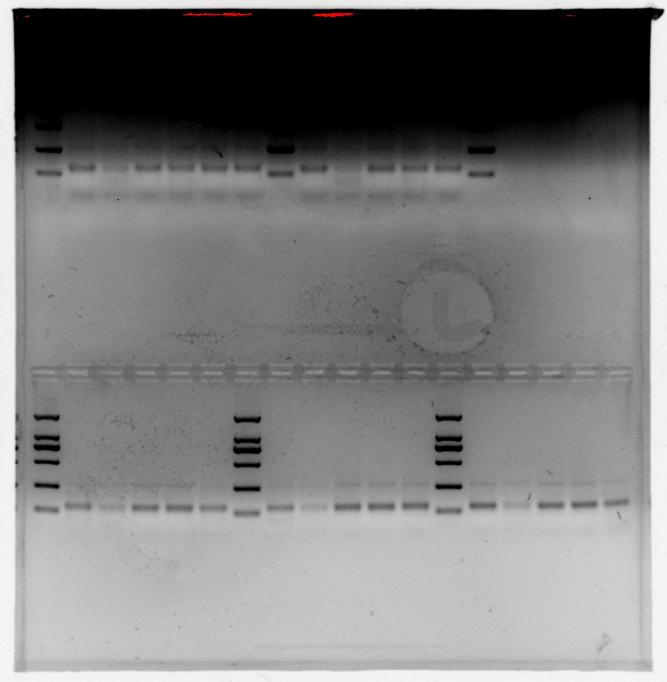


5G_CDKN1A


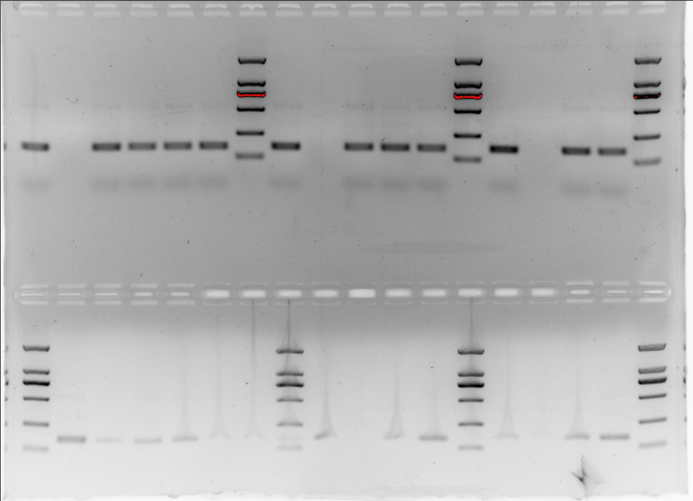


5G_CDKN1B

**Figure 6**


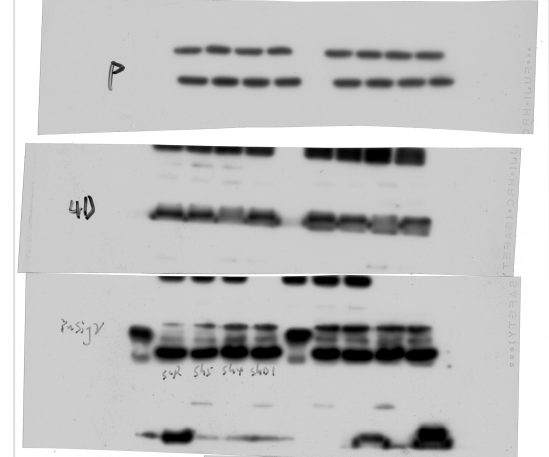


6C_INSIG2

6C_β-actin


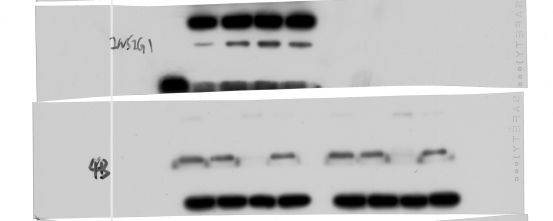


6C_INSIG1

6C_CUL4B


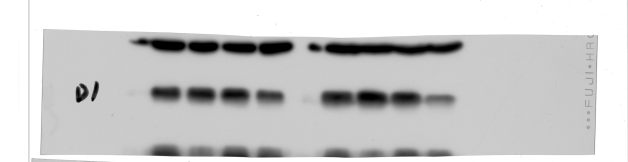


6C_DDB1

6C_KDM5B


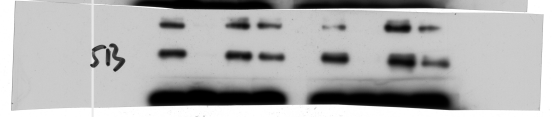


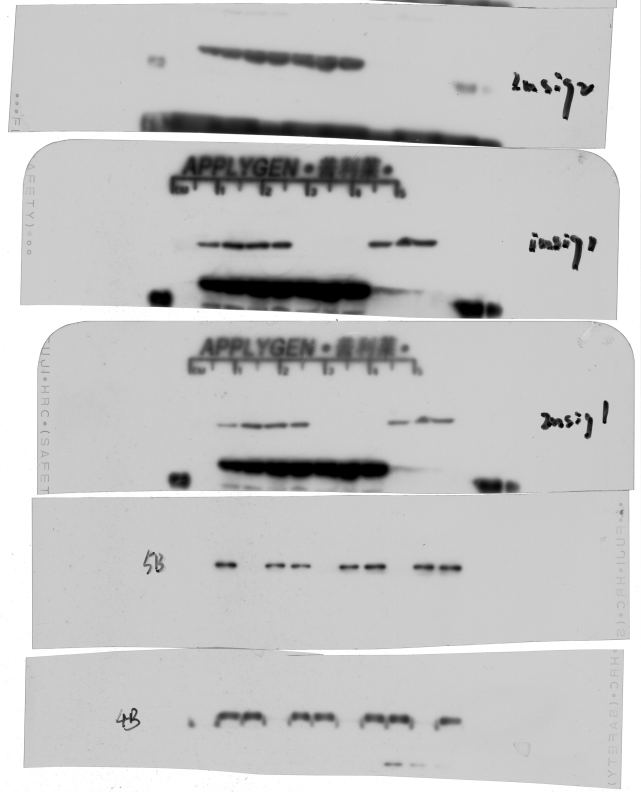


6E_INSIG2

6E_INSIG1

6E_KDM5B

6E_CUL4B


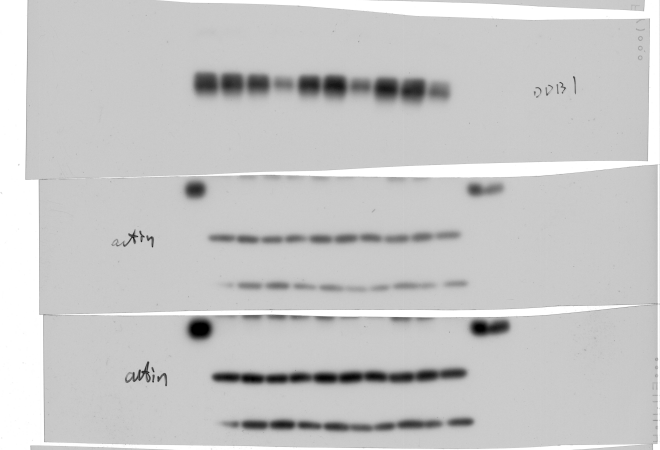


6E_DDB1

6E_β-actin


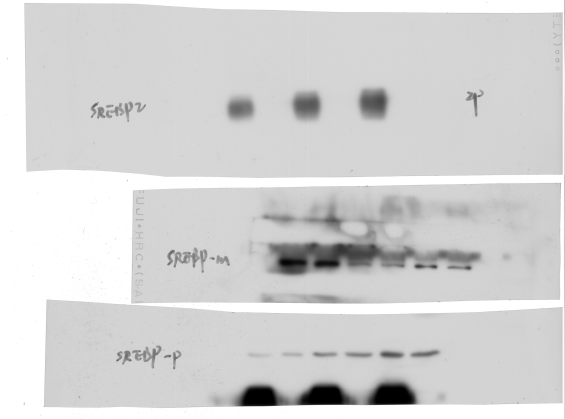

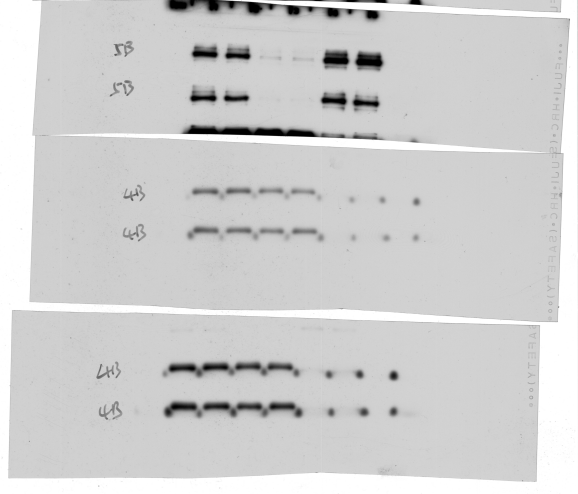

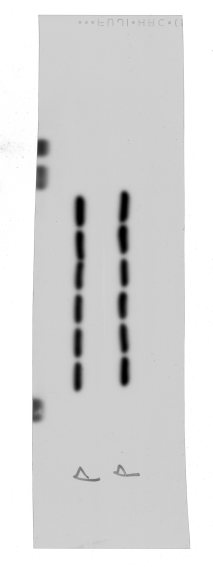


6I_β-actin

6I_KDM5B

6I_CUL4B

6I_SREBP2-P

6I_SREBP2-M_input

6I_SREBP2-P_input


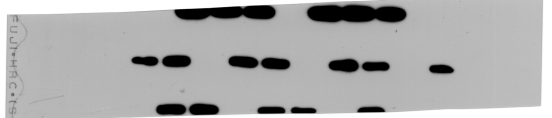

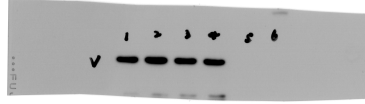


**Figure 7**


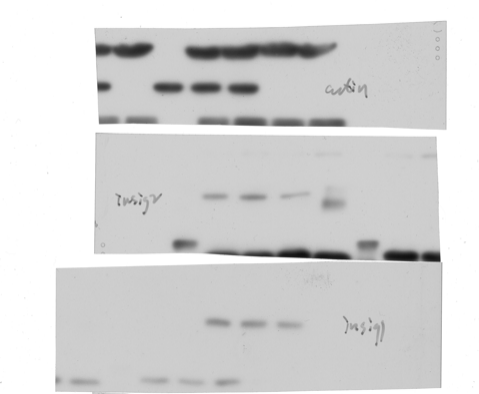


7D_MCF-7_actin

7D_MCF-7_INSIG1


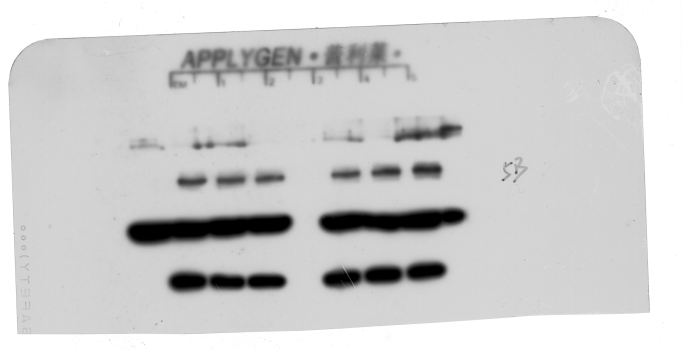


7D_MCF-7_KDM5B


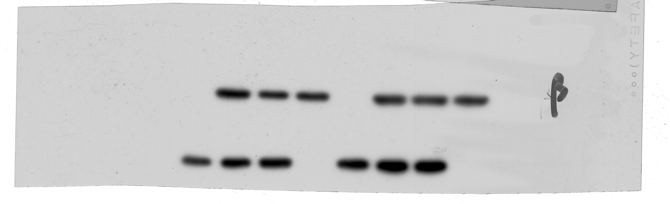

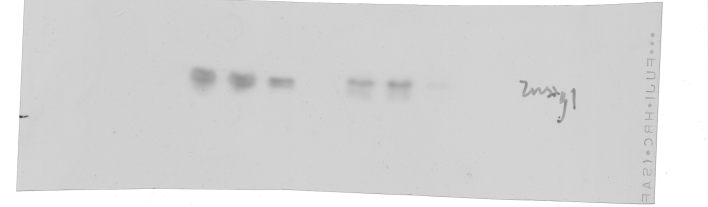

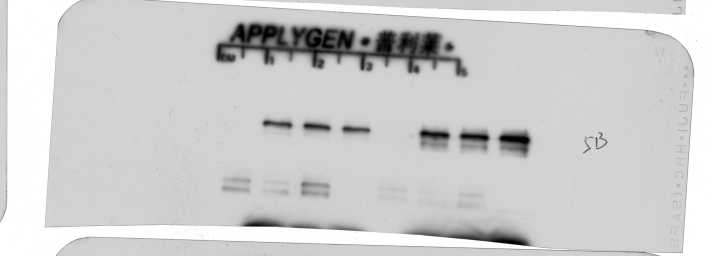


7D_T-47D_INSIG1

7D_T-47D_KDM5B

7D_T-47D_β-actin


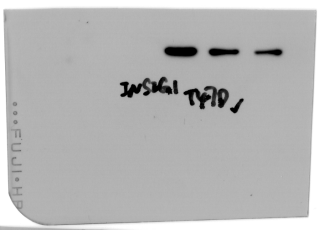

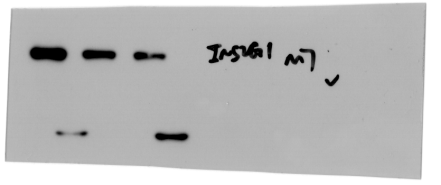


**Figure S3**


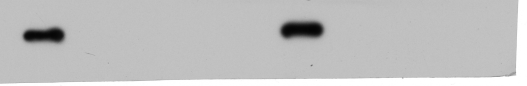

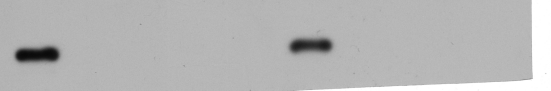


S3B_MCF7_KDM5B

S3B_T-47D_KDM5B


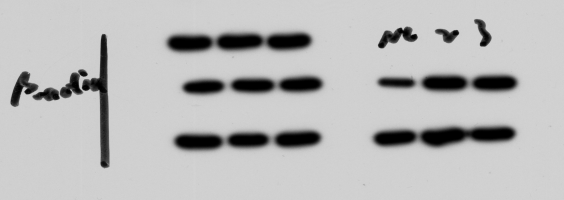


S3B_MCF7_β-actin

S3B_T-47D_β-actin


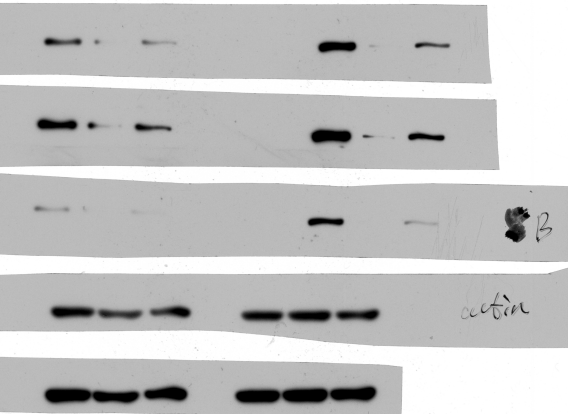

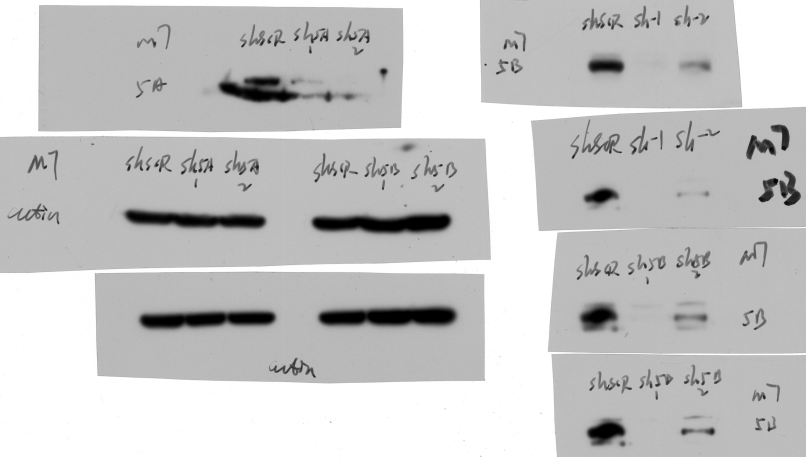


S3E_MCF7_KDM5B

S3E_T-47D_KDM5B

S3E_MCF7_β-actin

S3E_T-47D_β-actin


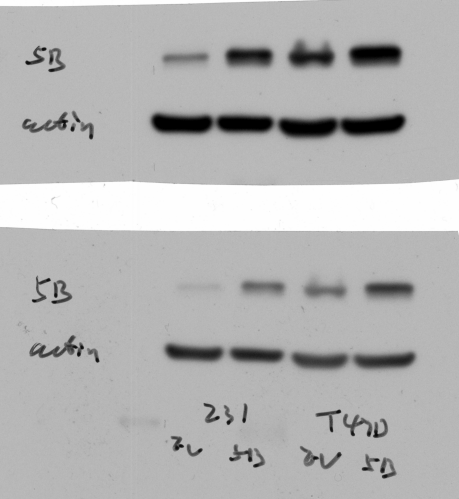


S3F


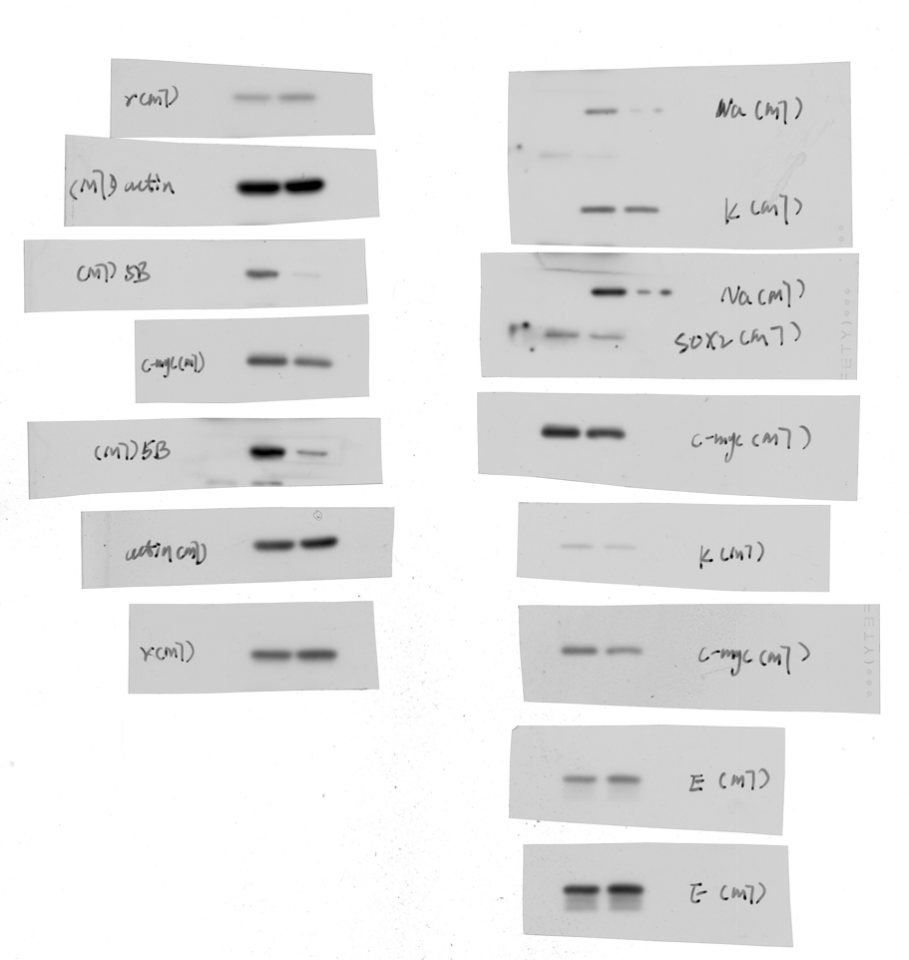

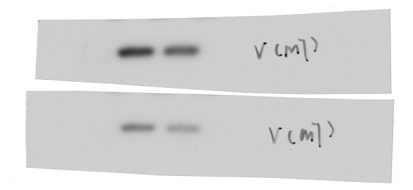


S3I_MCF7_KD_KDM5B

S3I_MCF7_KD_E-cadherin

S3I_MCF7_KD_Vimentin

S3J_MCF7_KD_NANOG

S3J_MCF7_KD_KLF4

S3J_MCF7_KD_SOX2

S3J_MCF7_KD_c-Myc

S3J_MCF7_KD_β-actin


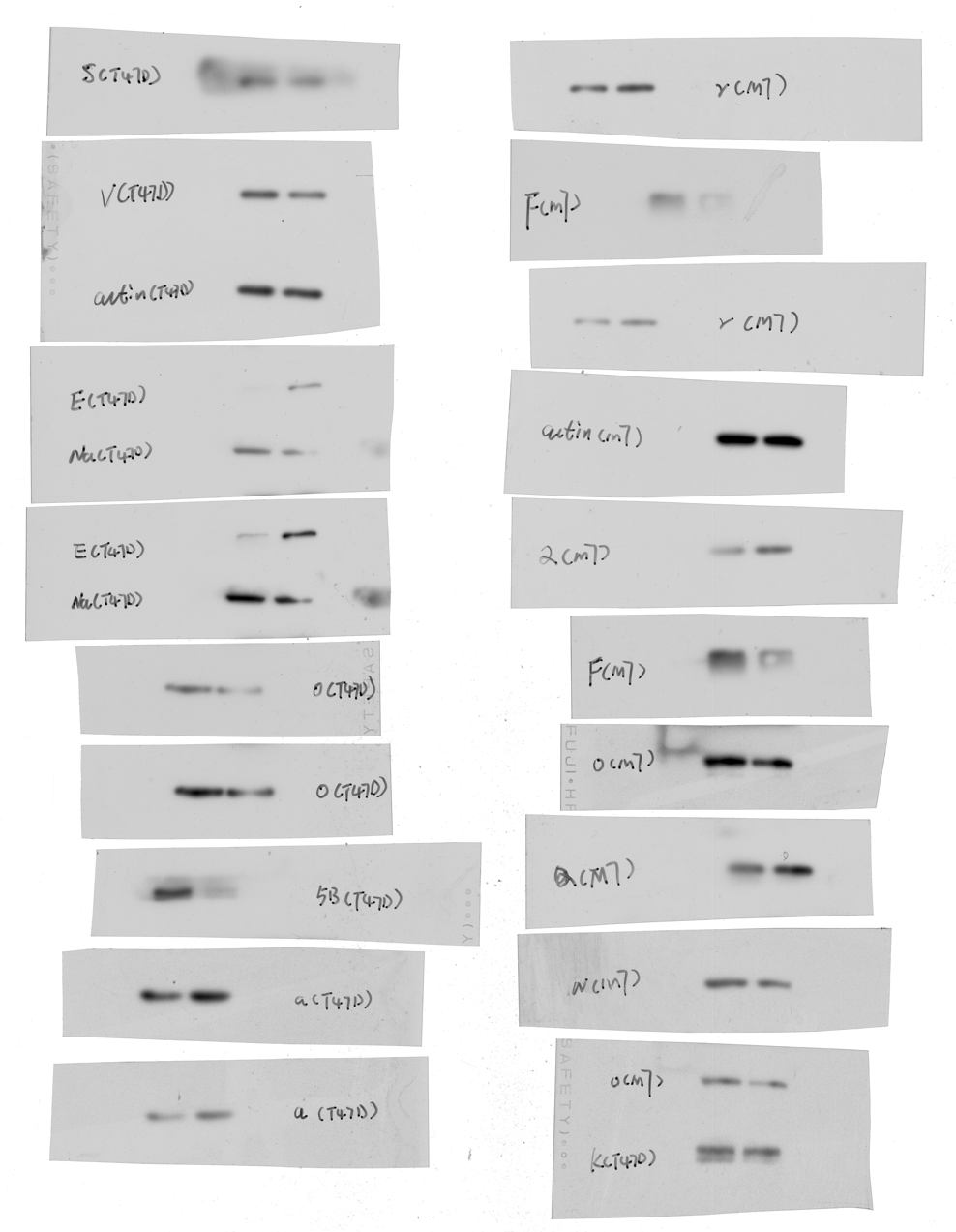


S3I_MCF7_KD_γ-catenin

S3I_MCF7_KD_α-catenin

S3I_MCF7_KD_β-actin

S3I_MCF7_KD_Fibronectin

S3I_MCF7_KD_N-cadherin

S3J_MCF7_KD_OCT4

S3I_T-47D_KD_Vimentin

S3I_T-47D_KD_β-actin

S3I_T-47D_KD_E-cadherin

S3I_T-47D_KD_KDM5B

S3I_T-47D_KD_α-catenin

S3J_T-47D_KD_OCT4

S3J_T-47D_KD_KLF4

S3J_T-47D_KD_NANOG

S3J_T-47D_KD_SOX2


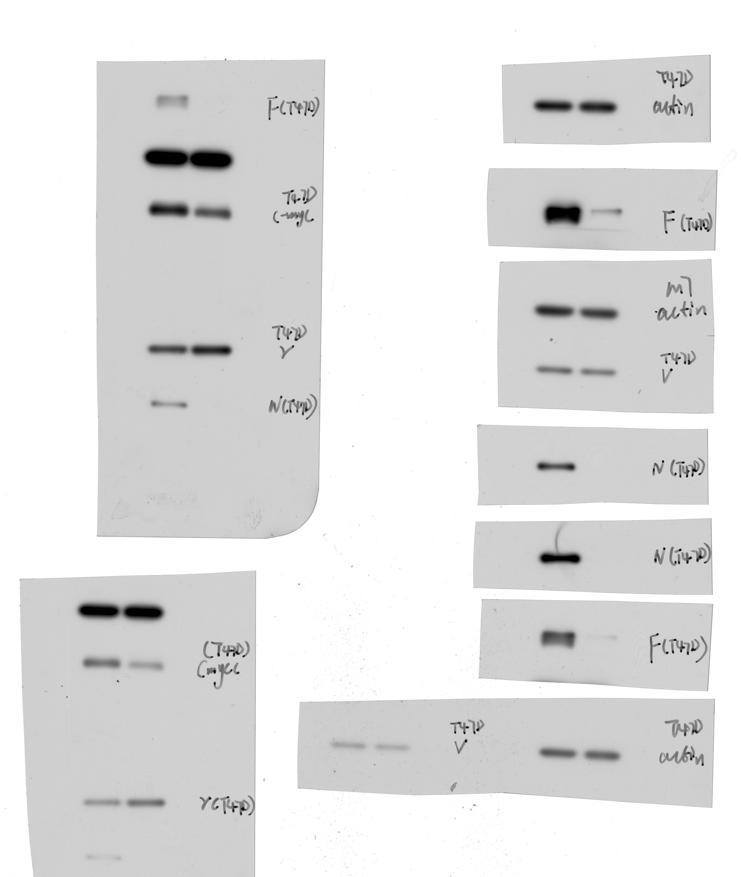


S3I_T-47D_KD_N-cadherin

S3I_T-47D_KD_γ-catenin

S3I_T-47D_KD_Fibronectin

S3J_T-47D_KD_c-Myc

S3J_T-47D_KD_β-actin


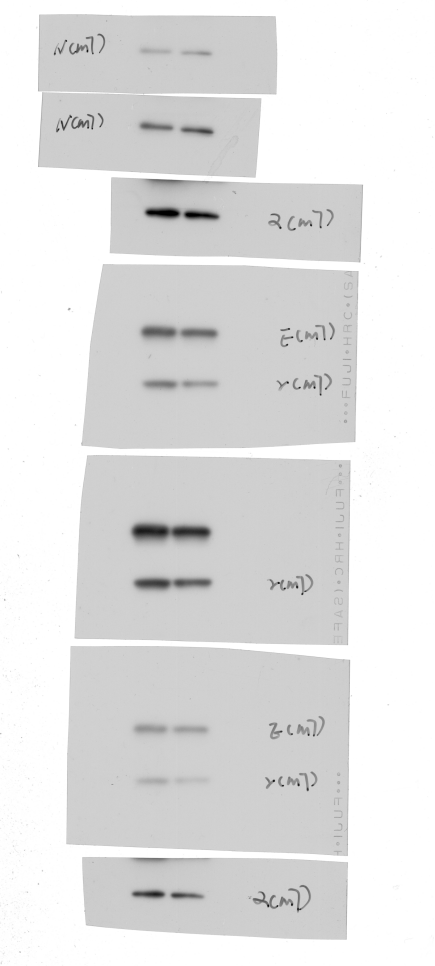

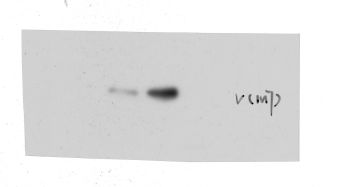

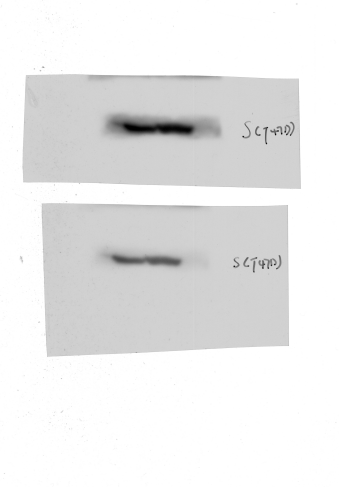


S3I_MCF7_OE_N-cadherin

S3I_MCF7_OE_α-catenin

S3I_MCF7_OE_E-cadherin

S3I_MCF7_OE_Vimentin

S3I_MCF7_OE_γ-catenin

S3J_T-47D_OE_SOX2


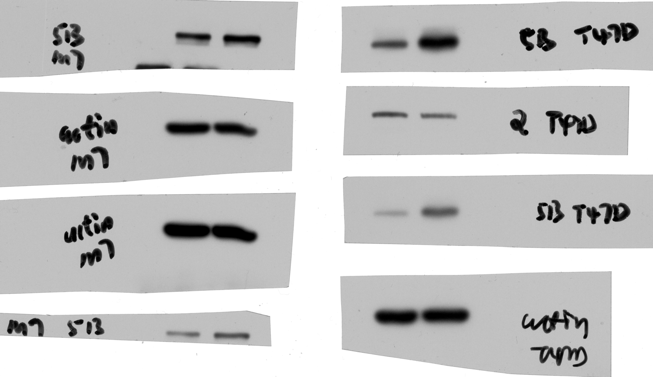


S3I_MCF7_OE_KDM5B

S3I_T-47D_OE_α-catenin

S3J_T-47D_OE_β-actin

S3J_T-47D_OE_KDM5B


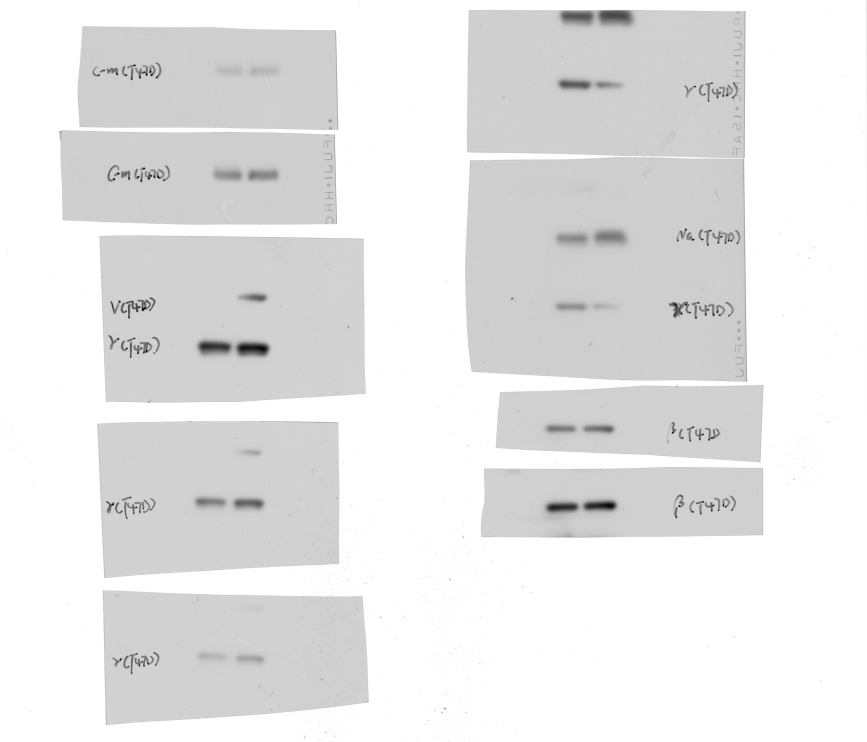


S3I_T-47D_OE_γ-catenin

S3I_T-47D_OE_Vimentin

S3J_T-47D_OE_NANOG

S3J_T-47D_OE_c-Myc


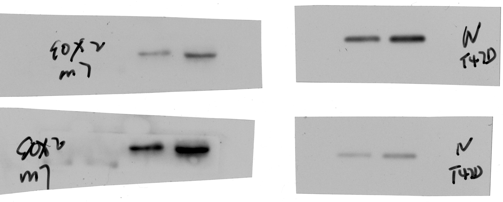


S3J_MCF7_OE_SOX2

S3I_T-47D_OE_N-cadherin


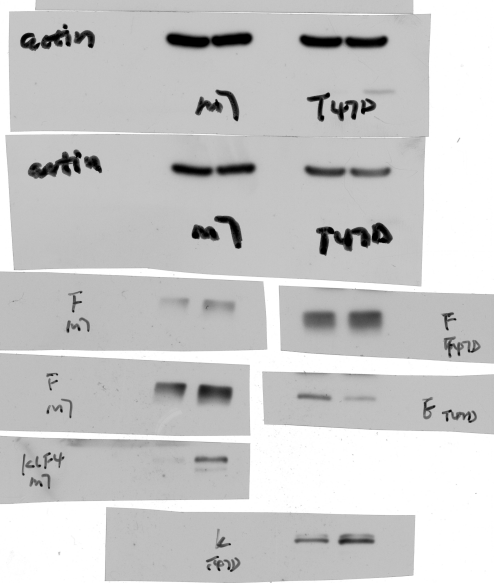


S3I_MCF7_OE_β-actin

S3I_T-47D_OE_β-actin

S3I_T-47D_OE_Fibronectin

S3I_MCF7_OE_Fibronectin

S3I_T-47D_OE_E-cadherin

S3J_T-47D_OE_KLF4

S3J_MCF7_OE_KLF4


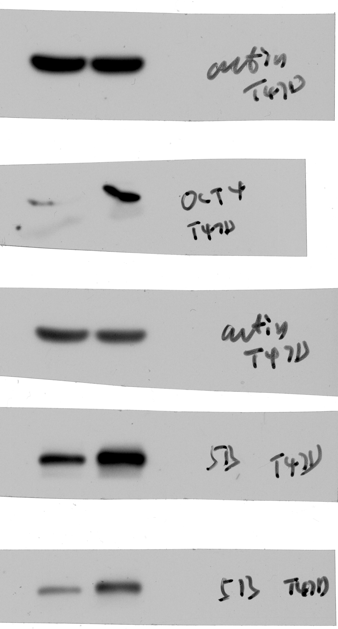

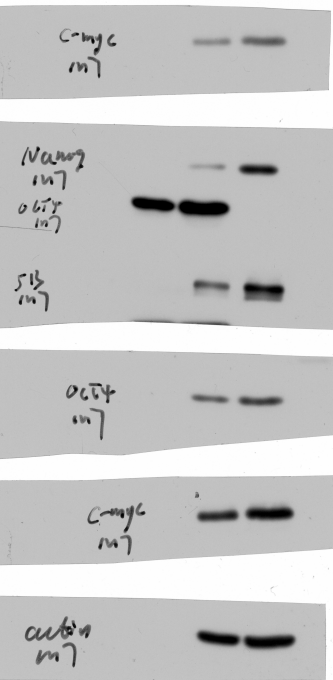


S3J_T-47D_OE_OCT4

S3I_T-47D_OE_KDM5B

S3J_MCF7_OE_c-Myc

S3J_MCF7_OE_NANOG

S3J_MCF7_OE_OCT4

S3J_MCF7_OE_KDM5B

S3J_MCF7_OE_β-actin


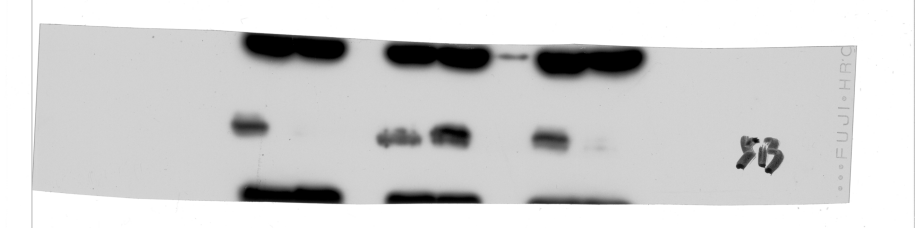


S3J_MCF-7_KD_KDM5B

S3J_T-47D_KD_KDM5B

**Figure S4**


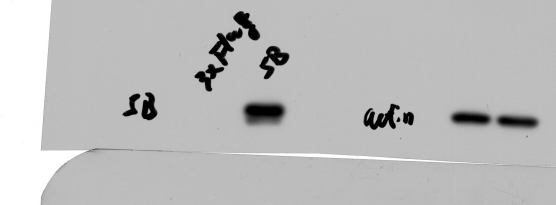

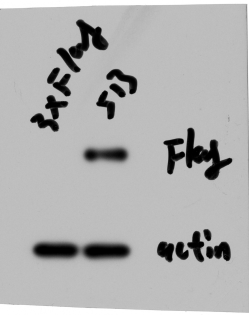


S4A_KDM5B

S4A_FLAG

S4A_β-actin


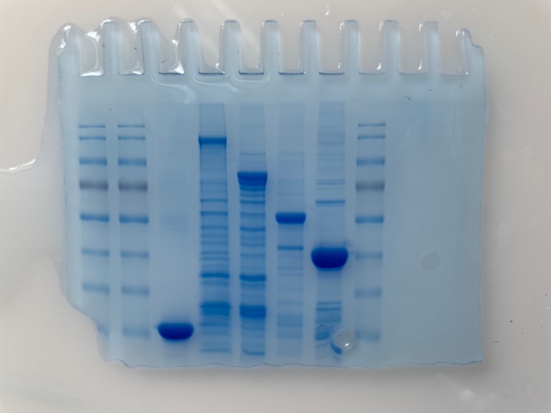

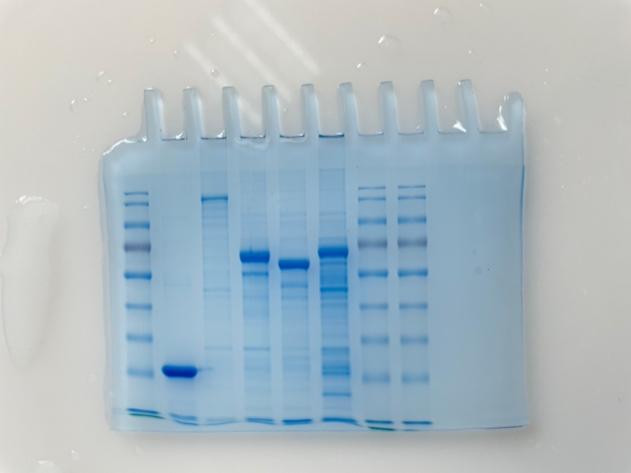

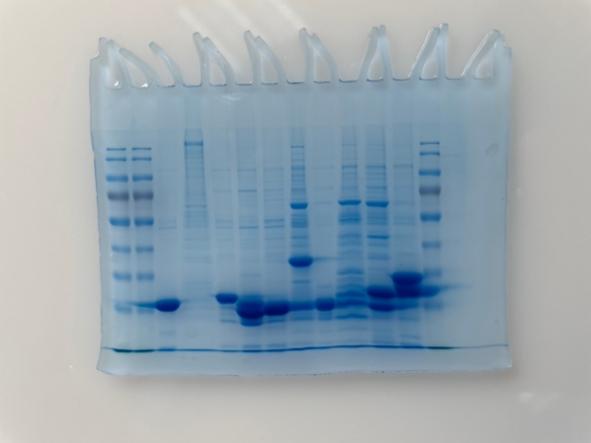


S4C

S4D_right

S4D_left

S4E_ROC1_3

S4E_ROC1_2

S4E_ROC1_1

S4E_CUL4B_2

S4E_CUL4B_3

S4E_CUL4B_1

S4E_DDB1_3

S4E_DDB1_2

S4E_DDB1_1

**Figure S5**

S5A_MCF7_KDM5B

S5A_MCF7_β-actin

S5A_T-47D_KDM5B

S5A_T-47D_β-actin

S5B_T-47D_β-actin

S5B_MCF7_β-actin

S5B_MCF7_KDM5B

S5B_T-47D_KDM5B

S5C_T-47D_oe_KDM5B

S5C_T-47D_oe_H3K4me3

S5C_MCF7_oe_H3K4me3

S5C_T-47D_oe_H3K4me2

S5C_MCF-7_oe_H3

S5C_T-47D_kd_H3K4me2

S5C_MCF-7_kd_H3K4me2

S5C_T-47D_oe_H3

S5C_MCF-7_kd_H3

S5C_MCF-7_oe_H3K4me2

S5C_T-47D_kd_H3K4me3

S5C_MCF-7_oe_actin

S5C_MCF-7_kd_actin

S5C_T-47D_kd_actin

S5C_MCF7_kd_H3K4me3

S5C_MCF7_oe_KDM5B

S5C_MCF-7_kd_KDM5B

S5C_T-47D_kd_KDM5B

S5C_T-47D_kd_H3

S5C_T-47D_oe_actin

**Figure S6**

S6B_MCF-7_KDM5B

S6B_231_actin

S6B_T-47D_actin

S6B_MCF-7_actin

S6D_SQLE

S6D_HMGCR

S6B_T-47D_KDM5B

S6B_231_KDM5B

S6B_1937_KDM5B

S6B_1937_actin

S6D_KDM5B

S6D_FDFT1

S6D_LDLR

S6D_actin

S6D_CUL4B
